# Voluntary activation of the ankle plantar flexors: A systematic review and meta-analysis

**DOI:** 10.1016/j.jsampl.2025.100117

**Published:** 2025-08-29

**Authors:** Molly E. Coventry, Christopher Latella, Brady Green, Andrea B. Mosler, Jayden Peak, Maria Luciana Perez Armendariz, Ebonie K. Rio, Myles C. Murphy

**Affiliations:** 1 Nutrition and Health Innovation Research Institute, School of Medical and Health Sciences, Edith Cowan University, Joondalup, WA, Australia; 2 Centre for Precision Health, Edith Cowan University, Joondalup, WA, Australia; 3 Neurophysiology Research Laboratory, School of Medical and Health Sciences, Edith Cowan University, Joondalup, WA, Australia; 4 School of Health Sciences, The University of Notre Dame, Fremantle, WA, Australia; 5 School of Allied Health, Human Services and Sport, La Trobe University, Melbourne, VIC, Australia; 6 LaTrobe Sport and Exercise Medicine Research Centre, La Trobe University, Bundoora, VIC, Australia; 7 Australian International Olympic Committee, La Trobe University, Bundoora, Victoria, Australia; 8 The Australian Ballet, Melbourne, VIC, Australia; 9 The Victorian Institute of Sport, Melbourne, VIC, Australia; 10 Institute for Health Research, The University of Notre Dame Australia, Fremantle, Western Australia, Australia

**Keywords:** Interpolated twitch, Nerve stimulation, Central activation ratio, Gastrocnemius, Soleus, Calf muscle

## Abstract

**Background:**

Voluntary activation is a measure of neural drive, typically measured during maximal contractions, and provides insight into motor function. This systematic review examined voluntary activation assessment of the ankle plantar flexors in healthy and pathological populations, and the association of participant age and positioning (knee and ankle joint angles) with voluntary activation level.

**Methods:**

A systematic review and meta-analyses were conducted. Six electronic databases were systematically searched for studies that assessed voluntary activation of the ankle plantar flexors using the interpolated twitch technique or the central activation ratio. Meta-analyses were performed using an inverse variance, random-effects maximal likelihood model of continuous outcomes within SPSS Statistics, and subsequent meta-regression performed for age, knee angle and ankle angle. Methodological quality was assessed using the Analytical Cross-Sectional Studies Checklist from the Joanna Briggs Institute.

**Results:**

A total of 74 studies were included, 5 included participants with pathological conditions (2 Achilles tendinopathy and 3 stroke) and 69 included only healthy participants. The meta-analysis demonstrated plantar flexion voluntary activation levels for healthy populations of 91 %, 90 % for Achilles tendinopathy and 35 % for stroke. Older age was associated with lower voluntary activation (β ​= ​−0.072; p ​= ​0.035) and greater knee flexion angle was associated with higher voluntary activation (β ​= ​−0.033; p ​= ​0.045). No association of voluntary activation and ankle position was demonstrated (β ​= ​−0.070, p ​= ​0.488). Majority of the included studies were judged to have low methodological quality (97 %).

**Conclusions:**

Voluntary activation was comparable between healthy participants and Achilles tendinopathy, and significantly lower in people following stroke. Age and knee joint position but not ankle joint position was associated with the level of plantar flexor voluntary activation.

**Key points**

1. Plantar flexion voluntary activation is comparable in healthy individuals and Achilles tendinopathy, but significantly lower following stroke.
2. One year of older age in adults is associated with 0.07 % lower voluntary activation in the ankle plantar flexors.
3. Higher knee flexion is associated with higher voluntary activation: 90° greater knee flexion is associated with 3 % higher voluntary activation.

## 1 Introduction

Soleus and gastrocnemius are the primary ankle plantar flexors [1]. They produce vertical and horizontal propulsion during walking and running [2] and also contribute to sport-specific and dynamic activities such as hopping/jumping, acceleration, deceleration and change direction [[3], [4], [5]]. The large work demands of these may contribute to the susceptibility of the ankle plantar flexors to muscle and tendon injuries [6,7]. From these perspectives, evaluating plantar flexion strength has gained recent attention in prevention and management [8]. However, peripheral muscle characteristics (e.g., muscle size, fat content, fibre profile) are not the only determinants of strength, other factors (e.g., the nervous system) may warrant further investigation. The motor nervous system underpins the ability to activate skeletal muscle and produce force [9]. During voluntary contractions, alterations in descending neural drive from the brain's primary motor cortex may contribute to reduced force generating capacity of skeletal muscle(s). In both clinical practice and research, muscular strength is often assessed with the performance of a maximal voluntary contraction (MVC) against manual resistance or equipment (e.g., dynamometers). However, standard MVC assessment lacks the ability to quantify the neural drive component of muscle strength.

Voluntary activation testing assesses the level of neural drive to the muscle during maximal force production and may provide functional insight into the motor nervous system [9]. The twitch interpolation technique is considered the gold standard and widely used to quantify neural drive [10], however, other methods such as the central activation ratio exist [11]. For the former, a superimposed supramaximal electrical stimulus (or multiple stimuli) is/are applied to the motor nerve during maximal voluntary contraction (usually isometric) and again, several seconds later with the muscle at rest. The size of superimposed twitch in relation to the twitch elicited at rest is used to quantify the level of voluntary activation (100 % indicating complete activation) [12]. Regardless of assessment technique, if additional force is created from superimposed stimulation, then it is implied that not all motor units were voluntarily activated by the individual, or that their activation was insufficient to produce maximal force [13].

Voluntary activation of the ankle plantar flexors have been assessed previously in healthy and pathological populations [14,15]. However, there appear to be numerous methodological discrepancies between studies, such as stimulation parameters, equipment used and participant positioning. Specifically, the influence of knee and ankle position on plantar flexion voluntary activation has been assessed by several studies with small sample sizes, and the results are inconsistent [[16], [17], [18], [19], [20], [21]]. Therefore, the impact of different testing position during the assessment of voluntary activation requires further investigation to guide clinical and research practices. Additionally, in some studies, age has been associated with voluntary activation levels, hence, we sought to explore whether meta-regression supported this association in plantar flexor voluntary activation [22]. Deficits in voluntary activation have also been linked to physical impairments in some pathological conditions in other joints (e.g., ACL reconstruction) [23]. The impairments in voluntary activation observed are typically small in magnitude but large in effect. Therefore, the impact of different methodological approaches for performing voluntary activation can have substantial influence on the interpretation of the data [24]. For this reason, our review aimed to explore how methodological variability between studies can influence plantar flexor voluntary activation.

This systematic review aimed to quantify maximal plantar flexion voluntary activation in healthy and pathological populations. We also aimed to evaluate the association of age and participant positioning (knee and ankle joint angles) with the level of maximal plantar flexion voluntary activation.

## 2 Methods

A preliminary search on PubMed and PROSPERO for previous and/or ongoing reviews on this topic was conducted and no similar reviews were revealed. This systematic review followed the Preferred Reporting Items for Systematic Reviews and Meta-Analyses (PRISMA) [25]. The review protocol was prospectively registered (PROSPERO registration number: CRD42024524305).

### 2.1 Inclusion criteria

#### 2.1.1 Participants

Humans aged 18 years and older irrespective of being either a case (any pathological condition) or control (non-pathological) participant were included. Inclusion was not restricted based on any other demographic features.

#### 2.1.2 Outcomes

The primary outcome was voluntary activation assessment of the ankle plantar flexors. Studies that reported any measure inclusive of: (i) voluntary activation; (ii) superimposed twitch torque (SIT); (iii) resting twitch torque (RT); (iv) central activation ratio (CAR); and, (v) central activation deficit (CAD) were included in full text review.

#### 2.1.3 Types of studies

Case-control, cross-sectional, cohort or longitudinal studies with voluntary activation outcomes were included. Randomised control trials (RCTs) that reported voluntary activation at baseline were also included. Studies were included regardless of their publication status. Case series, case reports and literature reviews were excluded. Studies published in languages other than English were translated where possible.

### 2.2 Search strategy

Search strategies were performed from inception to 14th March 2024 by one study author (MEC). Records were downloaded into EndNote and exported into Covidence.

#### 2.2.1 Electronic searches

Searches using free text terms (Supplementary file A) were performed within the following electronic databases; CINAHL (Full-text), SPORTDiscus, PubMed, Cochrane library, EBSCO (Medline) and Web of Science (Supplementary file B). Proquest was used to search for unpublished research within academic theses. Additional studies not found by database search were hand searched for within reference lists of reviews and retrieved articles. Studies yet to be indexed were identified for inclusion by screening the ePublication lists of key journals in the field.

### 2.3 Study selection

Studies identified by the search were exported to EndNote 20 [26] and uploaded into Covidence [27]. Covidence automatically removed all duplicates it could identify. Any duplicates not identified by Covidence were removed by reviewers during manual screening. A single reviewer (MEC) also checked the automatically removed duplicates to avoid erroneous exclusion. A single reviewer (MEC) paired with a second reviewer (either MCM, CL, BG, ABM or EKR) independently screened all titles and abstracts for inclusion against the preset inclusion criteria. The full text of studies that met the inclusion criteria were screened independently by two reviewers (MEC, MCM). Disagreements were resolved by discussion between reviewers until a consensus was reached.

### 2.4 Data extraction

A single author (MEC) paired with a second reviewer (either MLPA, LR and CA) independently extracted data from all included studies. All discrepancies were resolved by discussion between authors until a consensus was reached. The following data items were extracted: primary author; year of publication; country of study; study design; population; sport involvement (Yes/No); sample size (n); age (years); height (cm); weight (kg); sex (Male/Female/Intersex); comorbidities; force/torque equipment; EMG electrode placement; stimulation electrode placement; stimulation intensity; stimulus timing; joint angle (hip, knee and ankle); voluntary activation method; correct equation used (Yes/No); voluntary activation (%); maximal voluntary contraction force/torque (N/Nm). Knee and ankle joint angle measurements were standardised so that 0° represented 0 degrees of knee flexion (i.e., a straight knee) and a neutral ankle position (i.e., plantar grade). Where data was presented in a graphical format an online graph extractor tool was used to estimate values [28].

### 2.5 Data synthesis

#### 2.5.1 Data management

In studies that provided repeated voluntary activation values (e.g., multiple time points or pre/ post intervention), only baseline data was extracted. In non-interventional cross-over designs with no time point factor (e.g., studies exploring different testing positions), the mean and standard deviation were considered baseline data at all time points. For example, when healthy participants were randomised to either knee flexion or knee extension and then crossed-over, we included the group mean for each. In cases where voluntary activation was reported without an associated measure of variance, the missing variance was imputed using the median value derived from all studies that used an identical knee flexion angle [29]. Where the age was only reported as a range, the value in the middle of the range was imputed for the sample.

#### 2.5.2 Data analysis

Demographic data were described using count, percentage, mean and standard deviation. Voluntary activation data were sub-grouped for condition (i.e., Achilles tendinopathy versus stroke versus healthy control). Meta-analyses of between-group outcomes (i.e., Achilles tendinopathy versus stroke versus healthy individuals) were performed using an inverse variance, random-effects maximal likelihood model of continuous outcomes within SPSS Statistics (v.29.0, IBM, New York, USA). The meta-regression function within SPSS Statistics was used to evaluate, in separate regression models, any association of age, knee joint angle or ankle joint angle with voluntary activation.

#### 2.5.3 Sensitivity analysis

An a-priori sensitivity analysis for methodological quality had been planned. However, due to only two studies having high methodological quality, we deemed it inappropriate to perform this sensitivity analysis.

### 2.6 Assessment of methodological quality

Risk of bias was independently assessed by two authors (MEC and JP) using the Analytical Cross-Sectional studies check list from the Joanna Briggs Institute [30]. Any disagreements were resolved by consensus. The scale included eight items and with a defined a-priori criteria for each item (Supplementary file C). Methodological quality was assigned an overall judgement using a worst item counts basis. A tool designed for cross sectional studies was used as data from all studies were only extracted at a single time point, even if a study also included longitudinal outcomes.

### 2.7 Assessment of statistical heterogeneity

Heterogeneity was explored by the inclusion of relevant individual level covariates and factors (e.g., age) within meta-regression and statistical measures that describe how much variance (I^2^) exists within the model were reported. We considered statistical heterogeneity to be present when P < 0.10 or I^2^ ≥ 40 % [29].

### 2.8 Assessment of reporting biases

To account for small study bias, a risk of bias criterion based on study size was used. Studies including fewer than 50 participants were considered to have a high risk of bias, those with 50–200 participants were rated as moderate risk, and studies with more than 200 participants were deemed to have a low risk of bias [31]. Publication bias was assessed using the Eggers test within the SPSS Statistics meta-analysis function, with publication bias being evident when P < 0.05.

### 2.9 Assessment of the certainty of the body of evidence

The Grading of Recommendations, Assessment, Development and Evaluations (GRADE) approach was used to assess the quality of the body of evidence, as recommended in the Cochrane Handbook for Systematic Reviews of Interventions [29]. An overall judgement on the quality of the body of evidence is made based on the risk of bias, inconsistency, imprecision, indirectness and publication bias for each condition subgroup. Risk of bias was downgraded if the majority of studies were deemed as low risk of bias by the assessment of methodological quality criteria. Inconsistency was downgraded if statistical heterogeneity was present in the sample, demonstrated by P < 0.10 or I^2^ ≥ 40 %. Imprecision was downgraded for wide confidence intervals or confidence intervals which crossed zero and for small sample size. Publication bias was downgraded if the standard error coefficient in Egger's test is significantly different from zero. Indirectness was not judged in this review as it was not relevant to the meta-analyses.

## 3 Results

### 3.1 Selection of studies

**Fig. 1:**
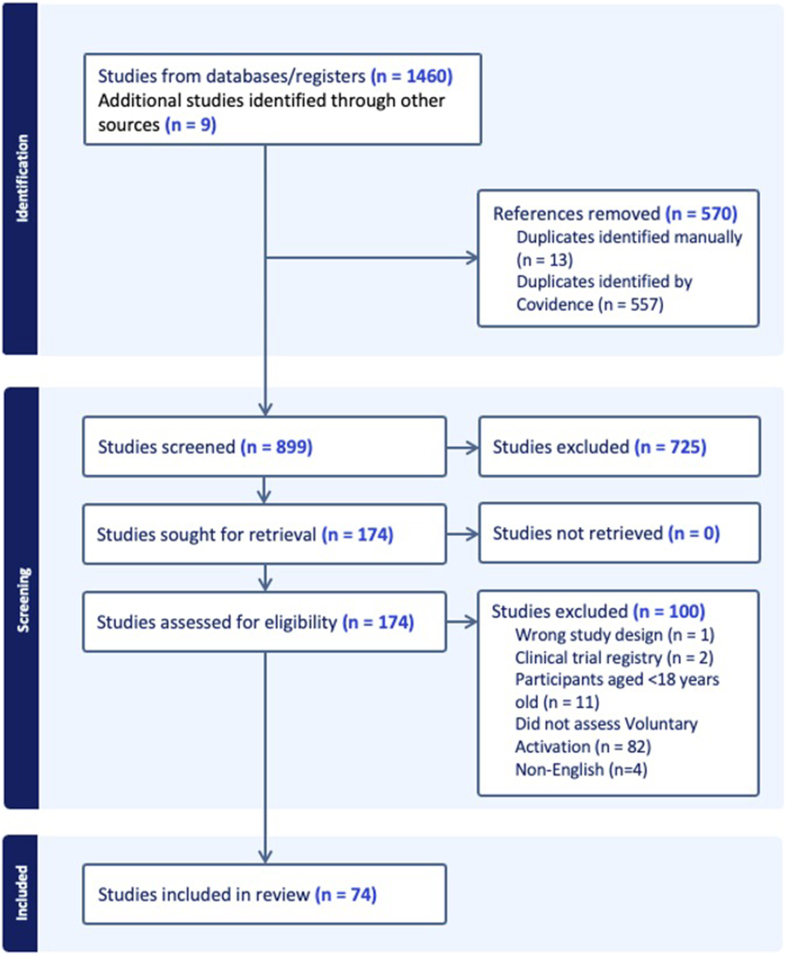
Prisma flowchart.

We identified 1469 studies, and after removal of duplicates and abstract screening, 173 records proceeded to full-text review. Seventy-four records were included in the final review (Fig. 1).

### 3.2 Study information

The 74 included studies were performed in 18 countries, 69 studies (93 %) included only healthy participants, two studies (3 %) compared participants with Achilles tendinopathy with healthy participants [32,33] and three studies (3 %) included only stroke participants [[34], [35], [36]]. In terms of study design, 43 % (32/74) were cross-sectional, 32 % (24/74) were quasi-experimental, 14 % (10/74) were cross-over trials, 9 % (7/74) were randomised control trials and the final study (1 %, 1/74) was a reliability study (Supplementary file D).

### 3.3 Participant demographics

Complete study level demographic details are provided (Supplementary file G). 1700 participants were included across all studies. The median study sample size was 14, ranging from 4 to 73 participants [37,38]. Females were included in 32 % (24/74) of studies, ranging from 1 to 22 participants and representing 23 % (390/1700) of the total cohort included in this review. No studies reported intersex participants. Mean age ranged from 20.4 to 84.0 years, mean height from 148 cm to 187 cm and mean body mass from 50.9 kg to 96.0 kg across groups in the included articles.

### 3.4 Participant position

The most common devices used to measure plantar flexion force/torque were an isokinetic dynamometer (54 %, 40/74) and a strain gauge (16 %, 12/74). The sampling rate at which force/torque was collected ranged from 500Hz to 10,000Hz, with the most common sampling rate used being 2,000Hz (45 %, 33/74). The participant position used to measure plantar flexion force/torque for each included study is detailed in Supplementary file E. The participant knee and ankle joint angles were reported in 98 % (73/74) of articles. Knee position ranged from 0 degrees of flexion (full extension) to 100 degrees of flexion. The knee was most frequently positioned in full extension (49 %, 36/74), followed by 90 degrees of flexion (30 %, 22/74). Ankle position ranged from 20 degrees of plantar flexion to 20 degrees of dorsiflexion. The ankle was most frequently positioned in neutral (80 %, 59/74), followed by 10° dorsiflexion (5 %, 4/74) and 20° dorsiflexion (4 %, 3/74). The most common combined position was full knee extension with a neutral ankle position (35 %, 26/74) followed by 90° knee flexion with a neutral ankle position (26 %, 19/74). 12 % (9/74) of articles assessed multiple knee and/or ankle joint positions.

### 3.5 Voluntary activation methodology

Electrical stimulation parameters for each included study are detailed in Supplementary file F. 95 % (70/74) of included studies used the interpolated twitch technique. The tibial nerve was stimulated in 93 % (69/74) of the articles and the calf muscle(s) (gastrocnemius or soleus) in 5 % (4/74). Regarding stimulation parameters, the most common wave shape used was a square (43 %, 32/74), followed by a rectangle (41 % 30/74). 12/74 (16 %) did not report stimulation shape. Single stimulation occurred in 46 % of studies (34/74) and doublet stimulation in 35 % (26/74). Stimulation duration ranged from 10μs to 1000μs, with 1000μs being the most frequently used (46 %, 34/74), followed by 100μs (20 %, 15/74). The stimulation intensity (i.e., milliamperes [mA]) used was only reported in 35 % (26/74) of articles, the means ranged from 76 mA to 378 mA. Supramaximal stimulation intensity was reported in 80 % (59/74) of articles and ranged from 110 % to 150 % of the intensity required to elicit a maximum response during set-up. 120 % was the most common intensity (39 %, 29/74).

### 3.6 Plantar flexion voluntary activation

Our meta-analysis demonstrated plantar flexion voluntary activation levels for healthy populations of 91.2 % (95 % CI = 89.8 to 92.6, I^2^ = 100 %) from 69 studies and 1629 participants, for Achilles tendinopathy 90.1 % (95 % CI = 74.1 to 106.7, I^2^ = 89 %) from two studies and 35 participants, and for stroke 35.4 % (95 % CI = 17.4 to 53.3, I^2^ = 32 %) from three studies and 36 participants. Voluntary activation was comparable between healthy participants and Achilles tendinopathy, and significantly lower in stroke (p < 0.001, Table 1).

**Table 1:** Expected voluntary activation effect sizes by population.

	Voluntary activation (%)	Standard Error	95 ​% Confidence Interval	
			Lower	Upper
Healthy	91.1	0.7	89.8	92.6
Achilles tendinopathy	90.1	8.3	74.1	100^a^
Stroke	35.4	9.1	17.4	53.3
Overall	90.7	0.8	89.1	92.2

a 95 ​% confidence interval upper limit is statistically 106.7, but imputed to 100 to reflect the maximal level of voluntary activation achievable.

### 3.7 Association between voluntary activation and age

Participant age was found to be associated with plantar flexion voluntary activation, whereby 1 year of additional age was associated with 0.072 % lower voluntary activation (95%CI = −0.139 to −0.005, p = 0.035, Table 2).

**Table 2:** Meta-regression of voluntary activation estimates by age, accounting for pathological group.

Parameter	Beta coefficient	95 ​% Confidence Interval		Significance (p)
		Lower	Upper	
(Intercept)	38.7	20.7	56.8	<0.001
Healthy	55.1^a^	37.2	72.9	<0.001
Achilles tendinopathy	54.9^a^	22.7	76.1	<0.001
Stroke	0^b^	.	.	.
Age	−0.072^c^	−0.1	−0.005	0.035

a Relative to stroke.

b This parameter is set to zero because it is redundant.

c The estimated voluntary activation change for each higher year of age.

### 3.8 Association between voluntary activation and knee joint angle

The degree of knee flexion during testing was significantly associated with plantar flexion voluntary activation, whereby 1-degree greater knee flexion angle was associated with 0.033 % higher voluntary activation (95%CI = 0.001 to 0.066, p = 0.045, Table 3). Summary statistics for each angle of knee flexion are displayed in Supplementary file H.

**Table 3:** Meta-regression of voluntary activation estimates by knee position, accounting for different pathological groups.

Parameter	Beta coefficient	95 ​% Confidence Interval		Significance (p)
		Lower	Upper	
(Intercept)	34.3	16.5	52.1	<0.001
Healthy	55.6^a^	37.8	73.5	<0.001
Achilles tendinopathy	56.9^a^	35.6	78.2	<0.001
Stroke	0^b^	.	.	.
Knee flexion	0.033^c^	0.001	0.066	0.045

a Relative to stroke.

b This parameter is set to zero because it is redundant.

c The estimated voluntary activation change for one degree higher knee flexion.

### 3.9 Association between voluntary activation and ankle joint angle

The degree of ankle dorsiflexion during testing was not associated with the level of plantar flexion voluntary activation (β = −0.070, 95%CI = −0.270 to 0.130, p = 0.488, Table 4).

**Table 4:** Meta-regression of voluntary activation estimates by ankle position, accounting for different pathological groups.

Parameter	Beta coefficient	95 ​% Confidence Interval		Significance (p)
		Lower	Upper	
(Intercept)	35.5	17.7	53.3	<00.001
Healthy	55.9^a^	38.0	73.7	<00.001
Achilles tendinopathy	55.7^a^	34.4	77.0	<00.001
Stroke	0^b^	.	.	.
Ankle dorsiflexion	−0.070^c^	−0.270	0.130	0.488

a Relative to stroke.

b This parameter is set to zero because it is redundant.

c The estimated voluntary activation change for one degree higher ankle dorsiflexion.

#### 3.9.1 Assessment of quality and bias in included studies

Our risk of bias assessment identified 97 % as low quality (72/74) and 3 % as high quality (2/74) (Supplementary file I). 86 % (64/74) studies had a sample size of <30 participants and were classified as high risk of small study bias. There was evidence of publication bias when evaluated using eggers regression statistic (Supplementary file J).

### 3.10 Certainty of the body of evidence

#### 3.10.1 Healthy populations

The certainty of the body of evidence was reported as ‘very low’ after being downgraded for risk of bias, inconsistency and publication bias (Supplementary file L).

#### 3.10.2 Achilles tendinopathy

The certainty of the body of evidence was reported as ‘very low’ after being downgraded for risk of bias, imprecision and inconsistency (Supplementary file L).

#### 3.10.3 Stroke

The certainty of the body of evidence was reported as ‘very low’ after being downgraded for risk of bias and imprecision (Supplementary file L).

## 4 Discussion

This systematic review and meta-analysis quantified the expected level of maximal voluntary activation of the ankle plantar flexors in healthy and pathological populations. Based on our meta-regression, ankle plantar flexor voluntary activation was ∼91 % in healthy populations, which was comparable to Achilles tendinopathy (∼90 %) and higher than stroke (∼35 %). Increased age and reduced knee flexion angle were associated with lower levels of plantar flexor voluntary activation.

Voluntary activation of the ankle plantar flexors in Achilles tendinopathy were found to be comparable to healthy participants, however the confidence intervals for the estimate are broad (95%CI = 74.1 to 100). The breadth of the confidence intervals for Achilles tendinopathy indicates substantial heterogeneity in the included Achilles tendinopathy research (I^2^ = 89 %). This heterogeneity may be due to a variety of reasons such as clinical or methodological variance and warrants further research. Our tendinopathy results contradict research in joint pathology such as anterior cruciate ligament reconstruction [39] and knee osteoarthritis [40] where substantial impairments in voluntary activation following injury were identified. However, since this judgement is based on only two low-quality studies with small sample sizes (n = 42 and n = 28), we have very low certainty in our estimate. In both studies, the participants had mild to moderate levels of disability, represented by mean scores of 65.6 % and 81.2 % of a patient reported outcome measure specific for Achilles tendinopathy. Therefore, future research is required to further explore if lower voluntary activation levels are associated with plantar flexor muscle injury.

Voluntary activation of the ankle plantar flexors was found to be substantially impaired following stroke in our meta-regression. However, as the confidence intervals are wide (95%CI = 17.4 to 53.3), we have low certainty in this estimate, likely attributed to the limited number of studies (n = 3). More broadly, we acknowledge that there is large clinical heterogeneity in stroke and related impairments and hence, these findings are unlikely generalisable across patients with different stroke sub-types, severities, lesion locations are associated factors. It is well established that there are deficits in voluntary activation of the paretic/more-affected elbow flexors [[41], [42], [43]], wrist flexors and extensors [41,44] and knee extensors [45,46] in comparison to the non-paretic/less-affected side and healthy controls. In a population of people with stroke, the results suggest the impairment of descending neural drive, particularly on the paretic/more-affected side, are substantial contributors to post-stroke voluntary muscle weakness in addition to peripheral factors (e.g., structural changes within the muscle). However, whilst we can be confident impairments to plantar flexion voluntary activation in stroke populations exist, due to the low certainty of the estimate in our meta-analysis, the true extent of the deficit across sub-types, injury severities and different durations (e.g., acute or chronic phase) is unknown.

Our meta-analysis indicated a relationship between older age and a reduced ability of the motor system to maximally drive muscles during maximal plantar flexion contractions. The meta-regression demonstrated that for every 10 years older in age, plantar flexor voluntary activation 0.72 % lower. These findings have clear clinical implications, as they suggest to maintain the same strength as younger adults, older adults’ plantar flexor muscles must work harder due to lower voluntary muscle activation. Such demands could have significant ramifications for elite athletic populations, where the contraction of every muscle fibre could influence competition success. Additionally, several studies have identified increased age as a risk factor for muscle strain injury [47,48]. Another systematic review identified similar age-related deficits in voluntary activation of the ankle plantar flexors, as well as other muscle groups including knee extensors and elbow flexors [22]. Hence, the decline in muscle force associated with aging may not be solely due to muscle properties (e.g., lower cross-sectional area). For example, alterations in the motor areas of the brain associated with aging (e.g., cortical thinning, changes in excitability) may decrease the ability to activate motor neurons and hence, skeletal muscle, or dysfunction of the motor neurons themselves may result in lower voluntary force production [49].

Our results highlight the inconsistencies in participant position (e.g., knee and ankle joint position) that exist when assessing plantar flexor voluntary activation. The results from the meta-regression demonstrated knee joint angle, but not ankle joint angle, was significantly associated with voluntary activation. Specifically, we estimate a 30-degree greater knee flexion angle to be associated with 1 % higher voluntary activation. Thus, there is an estimated 3 % difference in voluntary activation when the knee is tested in full extension in comparison to 90° flexion. However, this result is marginally significant, and the true effect size may be smaller as the confidence interval is barely above zero (95%CI = 0.001 to 0.066). Additionally, several studies have investigated the influence of knee joint position on voluntary activation, without finding significant differences between positions [[16], [17], [18],^20^]. Many factors, related to either study design or the force-angle relationship of the calf muscles, may contribute to the observed differences in voluntary activation level variability across knee joint positions. One example related to study design is the observed differences in voluntary activation across knee positions may be attributed to the increased system compliance (e.g., seat position). Specifically, in positions with the knee in full extension the twitch force may, to some extent, dissipate into the seat padding on the isokinetic dynamometer. However, in knee flexion positions, where the knee is secured by more rigid structures there is less compliance and the twitch may appear larger. Regarding the force-angle relationship, soleus will strongly contribute to plantar flexion irrespective of knee joint position. In contrast, gastrocnemius is at a mechanical disadvantage in positions of knee flexion and will contribute less to the overall plantar flexion force/torque generation in knee flexion [50]. Thus, testing in greater knee flexion is typically performed to disadvantage gastrocnemius and more specifically evaluate the soleus. These findings may be useful clinically if tailoring the test position to the injured triceps surae muscle.

Considerable methodological differences were found between studies that may influence the validity of voluntary activation assessment and create methodological heterogeneity between studies. A rigid setup is recommended as system compliancy may reduce the size of the superimposed and resting twitch [9]. 54 % of studies used an isokinetic dynamometer to assess plantar flexion voluntary activation. Despite being considered the gold standard for plantar flexion strength testing, compliancy in the system (e.g., padding/cushioning) potentially creates implications for collecting accurate superimposed twitch torques due to force absorption [9]. There is further discrepancy in the stimulation parameters used during the assessment. Stimulation duration, shape, stimuli per trial and intensity varied greatly between studies, with no clear majority in any parameter. Additionally, there is a concerning number of articles that did not report stimulation parameters at all. Some factors, such as the number of stimuli used, may not cause a significant difference in results [51]. However, the influence of other factors, such as stimulation duration and shape on voluntary activation is unclear. However, non-supramaximal stimulation intensities may reduce the validity of voluntary activation assessment. For example, sub-maximal stimulation intensities may not be sufficient to activate all motor neurons, and or surface stimulating electrode position in relation to the motor nerve may change during contraction resulting in altered twitch responses [9]. Both factors will likely artificially inflate the level of activation.

Our systematic review findings are limited by the overall methodological quality of the included studies. Based on our risk of bias judgement, 97 % of studies were judged as low quality, with 86 % of studies classed with a high risk of small study bias, demonstrated by the median sample size of 14 participants. The increased risk of bias was attributed to missing information on participant characteristics, methodological data (e.g., stimulation parameters) and confounding factors. There was also significant heterogeneity present within the sample, particularly in the healthy population which had an I^2^ value of 100 %. Our systematic review findings should be interpreted with consideration to the limitations in the quality of evidence and the low level of evidence available in pathological groups.

## 5 Conclusion

Our systematic review demonstrated ankle plantar flexion voluntary activation levels of ∼91 % in healthy people, which is comparable to Achilles tendinopathy (∼90 %) and significantly higher than in stroke (∼35 %). Our meta-analyses indicate that both age and knee joint position, but not ankle joint position, are associated with plantar flexor voluntary activation. When testing voluntary activation in clinical and research settings, it is important to consider knee, but not ankle, joint position in the testing set-up and participant age as a cofounding factor.

## CRedIT authorship contribution statement

**Molly Coventry:** Writing – original draft, Project administration, Methodology, Database search, Study screening, Data extraction, Quality appraisal, Formal analysis, Data curation. **Christopher Latella:** Writing – review & editing, Methodology, Study screening. **Brady Green:** Writing – review & editing, Methodology, Study screening. **Andrea Mosler:** Writing – review & editing, Methodology, Study screening. **Jayden Peak:** Writing – review & editing, Quality appraisal. **Maria Luciana Perez Armendariz:** Writing – review & editing, Data extraction. **Ebonie Rio:** Writing – review & editing, Methodology, Study screening. **Myles Murphy:** Writing – review & editing, Methodology, Study screening, Formal analysis, Supervision, Conceptualisation.

## Declaration of competing interest

None declared.
