# Supplementary material for: Voluntary activation of the ankle plantar flexors: A systematic review and meta-analysis
Source: JSAMS Plus. 2025 Aug 29;6:100117. doi: 10.1016/j.jsampl.2025.100117 (PMC13008425; doi:10.1016/j.jsampl.2025.100117)
Supplement: Multimedia component 1 [file mmc1.pdf]

Supplementary file A. Search strategy

Number	Combiners	Region	Terms
1	Problem of Interest	Title	Calf OR Gastrocnemius OR Soleus OR triceps surae OR Achilles OR Plantar flex*
2	Outcome	All	Voluntary activation OR Interpolated Twitch OR Superimposed twitch* OR Resting twitch* OR Central activation*
3			#1 AND #2
	Limitations		Human

Supplementary file B. Search Strategy Documentation

Source:	Date of search	Search strategy used (keywords & Boolean)	Search Limits (e.g., dates, language)	# results found	Comments
PUBMED	14/03/2024	(Calf[Title] OR Gastrocnemius[Title] OR Soleus[Title] OR triceps surae[Title] OR Achilles[Title] OR Plantar flex*[Title]) AND (Voluntary activation OR Interpolated Twitch OR Superimposed twitch* OR Resting twitch* OR Central activation*)	Humans	578	Exported to End Note
CINAHL (Full Text)	14/03/2024	TI (Calf OR Gastrocnemius OR Soleus OR triceps surae OR Achilles OR Plantar flex*) AND TX (Voluntary activation OR Interpolated Twitch OR Superimposed twitch* OR Resting twitch* OR Central activation*)	Humans	34	Exported to End Note
EBSCO (Medline)	14/03/2024	TI (Calf OR Gastrocnemius OR Soleus OR triceps surae OR Achilles OR Plantar flex*) AND TX (Voluntary activation OR Interpolated Twitch OR Superimposed twitch* OR Resting twitch* OR Central activation*)	Humans	104	Exported to End Note
Cochrane Library (including clinicaltrials.gov and International Clinical Trials Registry Platform)	14/03/2024	Calf OR Gastrocnemius OR Soleus OR triceps surae OR Achilles OR Plantar flex* in Record Title AND Voluntary activation OR Interpolated Twitch OR Superimposed twitch* OR Resting twitch* OR Central activation* in All Text - (Word variations have been searched)		106	Exported to End Note
SportsDISCUS	14/03/2024	TI (Calf OR Gastrocnemius OR Soleus OR triceps surae OR Achilles OR Plantar flex*) AND TX (Voluntary activation OR Interpolated Twitch OR Superimposed twitch* OR Resting twitch* OR Central activation*)		110	Exported to End Note
Web of Science	14/03/2024	TI (Calf OR Gastrocnemius OR Soleus OR triceps surae OR Achilles OR Plantar flex*) AND TX (Voluntary activation OR Interpolated Twitch OR Superimposed twitch* OR Resting twitch* OR Central activation*)		956	Exported to End Note
Proquest	14/03/2024	title(Calf OR Gastrocnemius OR Soleus OR triceps surae OR Achilles OR Plantar flex*) AND (Voluntary activation OR Interpolated Twitch OR Superimposed twitch* OR Resting twitch* OR Central activation*)		142	Exported to End Note
				TOTAL	2030

Supplementary file C. Quality assessment criteria

Item Number	Item Definition	Criteria for a judgement of 'YES'
1	Were the criteria for inclusion in the sample clearly defined?	Clear study inclusion criteria were defined, with sufficient detail to allow replication of the study. Study screened for musculoskeletal and neurological injury history.
2	Were the study participants and the setting described in detail?	Study subject characteristics (age, sex, body mass index, medical co-morbidities) and setting information were clearly defined and included sufficient detail to allow replication of the study.
3	Was the exposure measured in a valid and reliable way?	The study reported sufficient detail surrounding electrical stimulation protocol and used supramaximal stimulation of >120%.
4	Were objective, standard criteria used for measurement of the condition?	Where relevant, criteria used to measure the presence of pathology was clearly defined and used objective, standard criteria.
5	Were confounding factors identified?	Confounding factors, which may influence study results are identified in the study design.
6	Were strategies to deal with confounding factors stated?	Appropriate analysis employed to attempt to account for identified confounding factors.
7	Were the outcomes measured in a valid and reliable way?	Methods for voluntary activation and maximal isometric strength assessment were clearly described and were measured in a valid and reliable way.
8	Was appropriate statistical analysis used?	The statistical model used within the study was appropriate for their research question and a valid voluntary activation formula was used.

Supplementary file D. Individual study information

Study	*Country*	*Study design*	*Population*
Akagi et al. (a) (1)	Japan	Cross over	Healthy
Akagi et al. (b) (2)	Japan	Cross-sectional	Healthy
Avela et al. (3)	Finland	Quasi-experimental	Healthy
Barber et al. (4)	Australia	Cross-sectional	Healthy
Behm et al. (5)	Canada	Quasi-experimental	Healthy
Cannavan et al. (6)	England	Cross over	Healthy
Cattagni et al. (a) (7)	France	Cross-sectional	Healthy
Cattagni et al. (b) (8)	France	Quasi-experimental	Healthy
Crivelli et al. (9)	Switzerland	Cross over	Healthy
Crouzier et la. (10)	France	Cross-sectional	Achilles tendinopathy
Cruz et al. (11)	Brazil	Quasi-experimental	Healthy
Dalton et al. (a) (12)	Canada	Cross-sectional	Healthy
Dalton et al. (b) (13)	Canada	Cross-sectional	Healthy
Dalton et al. (c) (14)	Canada	Cross-sectional	Healthy
Dalton et al. (d) (15)	Canada	Cross-sectional	Healthy
Ekblom et al. (16)	Sweden	Randomised control trial	Healthy
Ema et al. (17)	Japan	Cross-sectional	Healthy
Fimland et al. (18)	Norway	Cross-sectional	Stroke
Girard et al. (a) *(19)*	France	Cross over	Healthy
Girard et al. (b) *(20)*	Switzerland	Quasi-experimental	Healthy
Gondin et al. (a) (21)	France	Randomised control trial	Healthy
Gondin et al. (b) (22)	France	Randomised control trial	Healthy
Green et al. (23)	New Zealand	Cross-sectional	Healthy
Grospretre et al. (24)	France	Cross-sectional	Healthy
Guette et al. (25)	France	Cross-sectional	Healthy
Hali et al. (26)	Canada	Cross over	Healthy
Hartman et al. (27)	USA	Cross-sectional	Healthy
Harwood et al. (28)	Canada	Quasi-experimental	Healthy
Herda et al. (29)	USA	Cross-sectional	Healthy
Hirata et al. (a) (30)	Japan	Randomised control trial	Healthy
Hirata et al. (b) (31)	Japan	Cross-sectional	Healthy
Hoffman et al. (32)	Australia	Quasi-experimental	Healthy
Hotta et al. (33)	Japan	Quasi-experimental	Healthy
Jo et al. (34)	Canada	Cross-sectional	Healthy
Kawakami et al. (35)	Japan	Cross over	Healthy
Kennedy et al. (36)	Canada	Quasi-experimental	Healthy

Kirk et al. (37)	Canada	Cross-sectional	Healthy
Klass et al. (38)	Belgium	Quasi-experimental	Healthy
Klein et al. (39)	Canada	Cross-sectional	Stroke
Knarr et al. (40)	USA	Cross-sectional	Stroke
Koryak et al. (a) (41)	Russia	Quasi-experimental	Healthy
Koryak et al. (b) (42)	Russia	Quasi-experimental	Healthy
Kubo et al. (a) (43)	Japan	Randomised control trial	Healthy
Kubo et al. (b) (44)	Japan	Cross-sectional	Healthy
Kuchinad et al. (45)	Canada	Quasi-experimental	Healthy
Lapole et al. (a) (46)	France	Cross over	Healthy
Lapole et al. (b) (47)	France	Quasi-experimental	Healthy
Maffiuletti et al. (48)	France	Quasi-Experimental	Healthy
Marathamuthu et al. (49)	Malaysia	Cross over	Healthy
Merlet et al. (50)	France	Reliability	Healthy
Miller et al. (51)	USA	Cross over	Healthy
Morse et al. (a) (52)	England	Cross-sectional	Healthy
Morse et al. (b) (53)	England	Cross-sectional	Healthy
Neyroud et al. (a) (54)	Switzerland	Cross-sectional	Healthy
Neyroud et al. (b) (55)	Switzerland	Quasi-experimental	Healthy
Nordlund et al. (56)	Sweden	Quasi-experimental	Healthy
Paris et al. (57)	Canada	Quasi-experimental	Healthy
Periard et al. (58)	Qatar	Cross over	Healthy
Phillips et al. (59)	USA	Cross-sectional	Healthy
Place et al. (60)	France	Cross-sectional	Healthy
Saldanha et al. (61)	Sweden	Cross-sectional	Healthy
Sara et al. (a) (62)	USA	Cross-sectional	Healthy
Sara et al. (b) (63)	USA	Quasi-experimental	Achilles tendinopathy
Scaglioni et al. (a) (64)	Italy	Quasi-experimental	Healthy
Scaglioni et al. (b) (65)	France	Quasi-experimental	Healthy
Scaglioni et al. (c) (66)	France	Cross-sectional	Healthy
Shimoda et al. (67)	Japan	Randomised control trial	Healthy
Siegler et al. (68)	Australia	Quasi-experimental	Healthy
Simoneau et al. (69)	France	Cross-sectional	Healthy
Stutzig et al. (70)	Germany	Quasi-experimental	Healthy
Suzuki et al. (71)	Japan	Cross-sectional	Healthy
Trajano et al. (72)	Australia	Randomised control trial	Healthy
Unhjem et al. (73)	Norway	Cross-sectional	Healthy
Weir et al. (74)	Canada	Quasi-experimental	Healthy

Supplementary file E. Individual study participant position

Study	Force transducer	Force sampling rate (Hz)	EMG	Knee joint position	Ankle joint position	Restraints
Akagi et al. (a) (1)	Isokinetic dynamometer	2000	MG, LG, SOL, TA	Full extension	Neutral	The participant's pelvis and ankle were secured on the reclining seat and the foot pedal adapter of the dynamometer with non-elastic straps and/or a seat belt.
Akagi et al. (b) (2)	Isokinetic dynamometer	4000	NR	Full extension	Neutral	The participant's torso, pelvic and ankle were secured on the reclining seat with elastic straps and seat belt
Avela et al. (3)	Ergometer chair	1000-3000	LG, SOL	60 degrees flexion	Neutral	NR
Barber et al. (4)	Isokinetic dynamometer	2000	MG, LG, SOL, TA	Full extension	Neutral	Custom adjustable foot restraint system secured foot to minimise heel lift from footplate during contractions
Behm et al. (5)	Modified boot apparatus	2000	SOL, TA	90 degrees flexion	Neutral	Legs secured in a modified boot apparatus
Cannavan et al. (6)	Isokinetic dynamometer	2000	MG, SOL, TA	Full extension	Neutral	Inelastic straps tightly placed across the chest, hips, thigh and foot
Cattagni et al. (a) (7)	Strain gauge	2000	SOL, MG, LG	Full extension	Neutral	Foot secured by straps
Cattagni et al. (b) (8)	Isokinetic dynamometer	2000	SOL, MG	20 degrees flexion	Neutral	NR
Crivelli et al. (9)	Strain gauge	2000	TA	90 degrees flexion	Neutral	Right foot secured with two Velcro bands to a custom footplate
Crouzier et la. (10)	Isokinetic dynamometer	1000	MG, LG, SOL	Full extension	Neutral	Foot was fixed with a rigid strap
Cruz et al. (11)	Load cell	2000	SOL	90 degrees flexion	Neutral	A padded limb support above the knee and an ankle- level belt were used during contractions.
Dalton et al. (a) (12)	Isokinetic dynamometer	2000	SOL, MG, TA	90 degrees flexion	10 degrees dorsiflexion	Participant was fastened securely to the seat of the Biodex with inelastic straps around the shoulders and waist. A thigh support with an inelastic strap was used for stabilization of the limb and to minimize involvement of the thigh muscles.

Dalton et al. (b) (13)	Isokinetic dynamometer	100	NR	90 degrees flexion and 10 degrees flexion	Neutral	Two Velcro straps across the toes and dorsum of the foot. Participants were fastened securely to the seat of the Biodex with inelastic straps around the shoulders and waist. A thigh support with an inelastic strap was used for stabilization of the limb and to minimize involvement of the thigh muscles.
Dalton et al. (c) (14)	Custom build isometric dynamometer	500	SOL, MG	90 degrees flexion	Neutral	Leg was secured with a C-clamp pressing firmly against the distal aspect of the right thigh to minimise extraneous hip and knee movement
Dalton et al. (d) (15)	Isokinetic dynamometer	100	NR	90 degrees flexion and 10 degrees flexion	Neutral	Foot was secured to the foot plate using a heel binding with an adjustable strap and participants were secured to the chair with straps around the shoulders and waist
Ekblom et al. (16)	Isokinetic dynamometer	5000	SOL	Full extension	Neutral	Shoulder pads and a broad Velcro strap over the right thigh of the participant were applied to prevent extraneous movements.
Ema et al. (17)	Isokinetic dynamometer	500	MG, LG, SOL, TA	Full extension and 90 degrees flexion	Neutral	Participants were secured at the body to the dynamometer with non- elastic straps
Fimland et al. (18)	Isometric dynamometer	2000	SOL, MG, LG, TA	Full extension	Neutral	Rigid straps made sure the heel and forefoot were secured to the footplate
Girard et al. (a) *(19)*	Dynamometric pedal	2000	SOL	90 degrees flexion	Neutral	The foot of the leg performing the MVC was secured to the dynamometric pedal with three restraining straps
Girard et al. (b) *(20)*	Dynamometric pedal	2000	NR	90 degrees flexion	Neutral	Foot securely strapped on the pedal by three straps
Gondin et al. (a) (21)	Strain gauge	2000	SOL, MG, LG	60 degrees flexion	Neutral	Foot secured to pedal
Gondin et al. (b) (22)	Isokinetic dynamometer	5000	SOL, MG, LG	90 degrees flexion	Neutral	To minimize hip and thigh motion during the contractions, and therefore to avoid the contribution of muscles other than the plantar flexors (e.g., knee extensors, hip flexors), straps were fastened across the chest and pelvis. The foot was also secured to the footplate by two straps. One strap was placed around the ankle and the second strap was placed around the

						foot, 1–2 cm proximal to the metatarsophalangeal joint of the toes.
Green et al. (23)	Custom build ergometer	NR	GM, TA	Full extension	Neutral	
Grospretre et al. (24)	Constraint gauge	2000	TA, VL, SOL, GM, GL	90 degrees flexion	Neutral	The ankle was firmly strapped to a pedal equipped with a constraint gauge
Guette et al. (25)	Strain gauge	2000	SOL, MG, LG, TA	60 degrees flexion	Neutral	Dominant foot was fixed to pedal by two straps.
Hali et al. (26)	Isokinetic dynamometer	500	TA, MG, LG, SOL	90 degrees flexion	20 degrees dorsiflexion and 20 degrees plantar flexion	The foot was secured to the dynamometer using two inelastic straps across the toes and dorsum of the foot and one at the ankle. A metal C-shaped bar pressing firmly against the distal aspect of the thigh minimized extraneous leg and hip movement during the contractions.
Hartman et al. (27)	Isokinetic dynamometer	2000	MG, SOL	Full extension	Neutral	Restraining straps over the pelvis, trunk and thigh. Foot was secured in a heel cup attached to a footplate with toe and ankle straps over the metatarsals and malleoli
Harwood et al. (28)	Linear calibrated force transducer	500	SOL, LG	90 degrees flexion	10 degrees plantar flexion	the foot firmly secured with restraints to the foot plate of the dynamometer.
Herda et al. (29)	Load cell	2000	SOL, MG	Full extension	NR	Restraining straps over the pelvis and thigh
Hirata et al. (a) (30)	Isokinetic dynamometer	2000	NR	Full extension	Neutral	pelvis and ankle were secured on the dynamometer bed and the foot pedal adapter of the dynamometer with non-elastic straps and/or a seat belt, respectively
Hirata et al. (b) (31)	Isokinetic dynamometer	2000	SOL, MG, LG	NR	Neutral	The right foot was fixed to the dynamometer foot plate with nonelastic straps
Hoffman et al. (32)	Torque transducer	2000	SOL, MG	5 degrees flexion	Neutral	The right foot strapped to a foot plate that was attached to a torque transducer.
Hotta et al. (33)	Load cell	1000	NR	Full extension	10 degrees plantar flexion	A belt was used as a support to keep the hip joint, knee joint, and thigh unchanged during testing. The foot was also tightly secured by two straps to keep the ankle joint unchanged.

Jo et al. (34)	Isokinetic dynamometer	NR	NR	Full extension	Neutral	Feet, chest and thighs were secured with non-elastic straps
Kawakami et al. (35)	Electrical myometer	1000	SOL, MG, LG	Full extension and 90 degrees flexion	10 degrees dorsiflexion	Waist, shoulders, trunk and thighs secured by Velcro bands
Kennedy et al. (36)	Isokinetic dynamometer	4000	SOL, MG, LG	Full extension	10 degrees plantar flexion	The torso, waist and right thigh were strapped to the dynamometer chair to ensure that the participant's body position did not change throughout the experiment.
Kirk et al. (37)	isometric dynamometer	500	SOL, MG, LG, TA	Full extension	Neutral	Two Velcro straps over the dorsum of the foot and c clamp pressed firmly against the distal aspect of the thigh
Klass et al. (38)	Strain gauge	2500	SOL, MG	Full extension	Neutral	The first strap was placed over the dorsum of the foot, and the second one was attached around the ankle and the calcaneum.
Klein et al. (39)	Custom built dynamometer	500	TA, LG, MG, SOL	90 degrees flexion	10 degrees dorsiflexion	The knee was stabilized from above with a U-shaped clamp. The foot was secured on the steel plate of the device with two Velcro straps: one across the instep, and the other 1 cm proximal to the great toe.
Knarr et al. (40)	Isokinetic dynamometer	NR	NR	Full extension	Neutral	Velcro straps were used to hold the foot and shank in position. Restraints were placed on the shoulders of the participant
Koryak et al. (a) (41)	Steal ring dynamometer	NR	NR	90 degrees flexion	Neutral	Firmly secured
Koryak et al. (b) (42)	Steal ring dynamometer	NR	SOL	90 degrees flexion	Neutral	the position of the seat was adjusted to the individual and then firmly secured. A rigid leg fixation ensured isometric conditions for the muscle contraction.
Kubo et al. (a) (43)	Isokinetic dynamometer	NR	MG, LG, SOL, TA	Full extension	Neutral	Waist and shoulders secured by adjustable lap belts
Kubo et al. (b) (44)	Electrical dynamometer	NR	NR	Full extension	Neutral	The waist and shoulders were secured by adjustable lap belts and were held in position
Kuchinad et al. (45)	Strain gauge	2500	SOL	90 degrees flexion	Neutral	An upright adjustable chair was used to support the subject, with a belt secured across the hips to keep the pelvis from shifting while seated. The lower limb was locked in place with

						an adjustable steel frame to eliminate compensatory movement during the experiment
Lapole et al. (a) (46)	Portable ergometer	2000	NR	20 degrees flexion	Neutral	Foot firmly strapped to footplate
Lapole et al. (b) (47)	Portable ergometer	NR	NR	60 degrees flexion	Neutral	
Maffiuletti et al. (48)	Strain gauge	2000	SOL, MG, LG, TA	90 degrees flexion	Neutral	Foot secured to pedal
Marathamuthu et al. (49)	Isokinetic dynamometer	2000	MG	Full extension	Neutral	Ankle secured with straps
Merlet et al. (50)	Isokinetic dynamometer	NR	SOL, GM	Full extension, 90 degrees flexion and 30 degrees flexion	Neutral	The participants were securely stabilized by two crossover shoulder harnesses and a belt across the abdomen.
Miller et al. (51)	Isokinetic dynamometer	2000	SOL	Full extension	15 degrees dorsiflexion and 20 degrees plantar flexion	Restraining straps over thigh, pelvis and trunk
Morse et al. (a) (52)	Isokinetic dynamometer	1000	TA	Full extension	20 degrees dorsiflexion	Straps were used about the hip to prevent forward displacement of the body
Morse et al. (b) (53)	Isokinetic dynamometer	NR	MG, LG	Full extension	15 degrees dorsiflexion, neutral and 20 degrees plantar flexion	Foot was secured tightly to footplate
Neyroud et al. (a) (54)	Strain gauge	2000	SOL, MG, LG, TA	Full extension	Neutral	Foot securely strapped at the level of the ankle and metatarsi to a pedal fixed to a wall. Straps were applied at the hip level.

Neyroud et al. (b) (55)	Strain gauge	2000	SOL, LG, MG	90 degrees flexion	Neutral	To limit the contribution of muscle groups other than plantar flexors, the thigh was clamped down to the chair proximal to the knee, while harnesses limited upper body movements.
Nordlund et al. (56)	Force transducer	5000	SOL, MG, TA	Full extension	Neutral	The pelvis was strapped to the bench to keep the knee in a straight position and prevent adverse movements during the protocol.
Paris et al. (57)	Isokinetic dynamometer	1000	NR	Full extension	10 degrees dorsiflexion	The right foot (self-reported dominant for all participants) was secured firmly to the footplate using an inelastic ratchet strap over the dorsum of the foot. Participants were further secured to the table using two large Velcro straps across the thighs and torso.
Periard et al. (58)	Dynamometric pedal	2000	NR	100 degrees flexion	Neutral	Foot was secured with two restraining straps
Phillips et al. (59)	Isokinetic dynamometer	1000	SOL, MG, LG	30 degrees flexion	Neutral	Restraining straps were placed over the chest, pelvis, and thigh,
Place et al. (60)	Isokinetic dynamometer	2000	SOL, MG, LG, TA	60 degrees flexion	Neutral	straps were fastened across the chest and pelvis. The foot was also secured to the footplate by two straps. One strap was placed around the ankle and the second strap was placed around the foot, 1–2 cm proximal to the metatarsophalangeal joint of the toes
Saldanha et al. (61)	Force transducer	1000	SOL	Full extension	Neutral	Foot was tightly strapped to a footplate
Sara et al. (a) (62)	Isokinetic dynamometer	NR	SOL, MG, LG, TA	90 degrees flexion	Neutral	Straps were placed across the waist, chest, and right thigh. Two straps were used around the ankle and one around the forefoot to maintain the plantar surface of the foot in contact with the foot plate
Sara et al. (b) (63)	Isokinetic dynamometer	NR	SOL, MG, LG, TA	Full extension	Neutral	Straps were placed across the waist, chest, and thigh. Two straps were secured around the ankle and one around the forefoot to maintain the plantar surface of the foot in contact with the foot plate

Scaglioni et al. (a) (64)	Isokinetic dynamometer	1000	SOL	Full extension	20 degrees dorsiflexion	The pelvis was firmly fixed to the examination table to prevent movements during efforts. A strap was placed around the foot to secure it sturdily to the strain gauge of the dynamometer
Scaglioni et al. (b) (65)	Strain gauge	2000	SOL	90 degrees flexion	Neutral	A strap was placed around the foot to secure it firmly to the pedal and thus create an effective mechanic constriction.
Scaglioni et al. (c) (66)	Custom made pedal with Strain gauge	2000	NR	90 degrees flexion	Neutral	A strap was placed around the foot to secure it firmly to the pedal and thus create an effective mechanic constriction.
Shimoda et al. (67)	Isokinetic dynamometer	1000	MG, LG SOL	Full extension	Neutral	Straps around the hips and right thigh to minimize excessive body motion
Siegler et al. (68)	Custom build ergometer	1000	SOL, MG	Full extension	5 degrees dorsiflexion	
Simoneau et al. (69)	Isokinetic dynamometer	2000	SOL, MG, LG, TA	Full extension	Neutral	Waist was stabilised by means of a belt and foot was secured with a strap
Stutzig et al. (70)	Plantar flexor device	2000	SOL, LG, MG	Full extension and 80 degrees flexion	Neutral	NR
Suzuki et al. (71)	Isokinetic dynamometer	10000	VL, GM, BF, TA, FL, MG, LG, SOL	Full extension	Neutral	Straps placed around the shoulders, hips, right knee, and left ankle to minimise changes in joint angles
Trajano et al. (72)	Isokinetic dynamometer		SOL, LG	Full extension	Neutral	NR
Unhjem et al. (73)	Force platform	2000	SOL, GM, GL, TA	90 degrees flexion	20 degrees dorsiflexion	NR
Weir et al. (74)	Strain gauge	6000	SOL, MG, TA	90 degrees flexion	Neutral	An adjustable knee clamp applied a downward pressure on the test limb, which eliminated lateral movements of the lower leg and prevented the heel from rising off the footplate during plantar flexion.

NR, not reported; EMG, electromyography; MG, medial gastrocnemius; LG, lateral gastrocnemius; SOL, soleus; TA, tibialis anterior; VL, vastus lateralis; BF, biceps femoris; VM, vastus medialis.

Supplementary file F. Individual study stimulation parameters

Study	Stimulation location	Stimulus shape	Stimulus type	Stimulus duration (μs)	Interstimulus interval (ms)	Stimulation intensity (mA)	Stimulation frequency (Hz)	Stimulus voltage (V)	Supramaximal stimulation intensity (% above maximum)	VA Method (ITT, CAR)
Akagi et al. (a) (43)	Tibial nerve in popliteal fossa	Rectangular	Triplet	200	10	NR	100	NR	20	ITT
Akagi et al. (b) (26)	Tibial nerve in popliteal fossa	Rectangular	Single	1000	-	NR	NR	NR	20	ITT
Avela et al. (44)	Tibial nerve in popliteal fossa	Rectangular	Doublet	1000	NR	NR	100	NR	25	ITT
Barber et al. (45)	Tibial nerve in popliteal fossa	Square	Doublet	100	10	20-150	NR	400	25	ITT
Behm et al. (46)	Superior aspect of gastrocnemius	Rectangular	Doublet	50-100	10	10-1000	NR	100	NR	ITT
Cannavan et al. (47)	Proximal and distal ends of the triceps surae	NR	Doublet	10	NR	NR	100	NR	40	ITT
Cattagni et al. (a) (48)	Tibial nerve in popliteal fossa	Rectangular	Single	1000	-	NR	NR	400	20	ITT
Cattagni et al. (b) (49)	Posterior tibial nerve in the popliteal fossa	NR	Doublet	1000	NR	NR	100	400	20	ITT
Crivelli et al. (50)	Tibial nerve in popliteal fossa	Square	Single and Doublet	1000	10	76(45)	NR	NR	10	ITT
Crouzier et la. (20)	Tibial nerve in popliteal fossa	NR	Doublet	1000	10	103.4(36.9)	NR	400	20	ITT
Cruz et al. (51)	Tibial nerve in popliteal fossa	Square	Single	200	-	NR	NR	400	30	ITT
Dalton et al. (a) (52)	Tibial nerve in popliteal fossa	Square	Single	100	-	378(92)	NR	400	10-15	ITT

Dalton et al. (b) (38)	Popliteal fossa	Square	Single	100	-	NR	NR	400	10-15	ITT
Dalton et al. (c) (53)	Tibial nerve in popliteal fossa	Square	Single	100	-	250-600	NR	400	10-15	ITT
Dalton et al. (d) (37)	Tibial nerve in popliteal fossa	Square	Single	100	-	240-850	NR	400	20	ITT
Ekblom et al. (54)	Tibial nerve in popliteal fossa	Square	Single	1000	-	NR	NR	NR	50	ITT
Ema et al. (39)	Tibial nerve in popliteal fossa	Rectangular	Single	1000	-	NR	NR	NR	20	ITT
Fimland et al. (24)	Tibial nerve in popliteal fossa	Square	NR	1000	NR	NR	NR	NR	100mA	ITT
Girard et al. (a) (55)	Tibial nerve in popliteal fossa	Rectangular	Single	200	-	85(22), 48-120	NR	400	50	ITT
Girard et al. (b) (56)	Tibial nerve in popliteal fossa	Rectangular	Single	200	-	130(31)	NR	400	50	ITT
Gondin et al. (a) (57)	Tibial nerve in popliteal fossa	Rectangular	Doublet	1000	10	NR	20-100	NR	NR	ITT, CAR
Gondin et al. (b) (58)	Posterior tibial nerve in popliteal fossa	Rectangular	Single	1000	-	32-120	75	NR	50	ITT
Green et al. (59)	Tibial nerve in popliteal fossa	Square	Single	100	-	90-210	NR	NR	20	ITT
Grospretre et al. (60)	Tibial nerve in popliteal fossa	Rectangular	Single	1000	-	NR	NR	NR	20	ITT
Guette et al. (61)	Posterior nerve in popliteal fossa	Rectangular	Doublet	1000	NR	0-100	NR	400	NR	ITT
Hali et al. (62)	Tibial nerve in popliteal fossa	Square	Single	200	-	60-120	NR	400	20	ITT
Hartman et al. (63)	Tibial nerve in popliteal fossa	Rectangular	Doublet	1000	NR	30-300	NR	NR	20	ITT, CAR
Harwood et al. (64)	Tibial nerve in popliteal fossa	Square	Doublet	100	NR	NR	NR	200	NR	ITT

Herda et al. (65)	Tibial nerve in popliteal fossa	Square	Doublet	200	NR	NR	100	400	20	ITT at 90% MVC
Hirata et al. (a) (66)	Tibial nerve in popliteal fossa	Rectangular	Triplet	200	10	NR	100	NR	20	ITT
Hirata et al. (b) (67)	Tibial nerve in popliteal fossa	Rectangular	NR	200	NR	NR	NR	NR	20	ITT
Hoffman et al. (68)	Popliteal fossa	NR	Doublet	NR	15	NR	NR	NR	20	ITT
Hotta et al. (69)	Tibial nerve in popliteal fossa	Square	Single	1000	-	NR	NR	NR	20	ITT
Jo et al. (70)	Tibial nerve in popliteal fossa	Rectangular	Single and Doublet	200	10	NR	NR	NR	10-15	ITT
Kawakami et al. (71)	Tibial nerve in popliteal fossa	Square	Doublet	500	10	NR	NR	NR	NR	ITT
Kennedy et al. (72)	Tibial nerve in popliteal fossa	NR	Single	200	-	173(30.5)	NR	NR	NR	ITT
Kirk et al. (73)	Tibial nerve in popliteal fossa	Square	Single	100	-	NR	NR	400	15	ITT
Klass et al. (74)	Tibial nerve in popliteal fossa	Rectangular	Single and Doublet	1000	10	NR	NR	NR	10-20	ITT
Klein et al. (22)	Tibial nerve in popliteal fossa	NR	Single	50-100	-	NR	25-100	NR	20	ITT
Knarr et al. (23)	NR	Square	Single	600	-	NR	100	135	NR	CAR
Koryak et al. (a) (75)	Tibial nerve in popliteal fossa	Rectangular	NR	1000	NR	NR	150	NR	30-40	NR
Koryak et al. (b) (25)	Tibial nerve in popliteal fossa	Rectangular	NR	1000	NR	NR	NR	NR	NR	NR
Kubo et al. (a) (76)	Tibial nerve in popliteal fossa	Rectangular	Triplet	500	10	NR	NR	NR	NR	ITT
Kubo et al. (b) (77)	Tibial nerve in popliteal fossa	Rectangular	Triplet	500	10	NR	NR	NR	NR	ITT
Kuchinad et al. (78)	Tibial nerve in popliteal fossa	Square	Doublet	50-100	NR	NR	100	NR	20	ITT

Lapole et al. (a) (79)	Tibial nerve in popliteal fossa	Rectangular	Single	1000	-	NR	NR	NR	25	ITT
Lapole et al. (b) (80)	Tibial nerve in popliteal fossa	NR	Single	1000	-	NR	NR	NR	20	ITT
Maffiuletti et al. (81)	Tibial nerve in popliteal fossa	Rectangular	Single	1000	-	NR	NR	NR	10	ITT
Marathamuthu et al. (82)	Tibial nerve in popliteal fossa	NR	NR	200	NR	45-120	NR	NR	20	ITT, CAR
Merlet et al. (40)	Tibial nerve in popliteal fossa	Rectangular	Single	1000	-	NR	NR	400	10	ITT
Miller et al. (83)	Tibial nerve	Square	Doublet	1000	NR	NR	100	NR	20	ITT
Morse et al. (a) (84)	Distal to the popliteal crease	Square	Doublet	50	10	NR	NR	NR	NR	ITT
Morse et al. (b) (85)	Tibial nerve in popliteal fossa	NR	Doublets	50	10	50-100	NR	NR	NR	ITT
Neyroud et al. (a) (86)	Tibial nerve in popliteal fossa	Rectangular	Single and Doublet	1000	NR	157(37), 120-228	NR	400	20	ITT
Neyroud et al. (b) (87)	tibial nerve	Rectangular	Doublet	1000	NR	NR	100	400	20	ITT
Nordlund et al. (88)	Tibial nerve in popliteal fossa	NR	Single	1000	-	NR	NR		50	ITT
Paris et al. (89)	Tibial nerve in popliteal fossa	Square	Single	100	-	89.5(17.8)	NR	400	20	ITT
Periard et al. (90)	Tibial nerve in popliteal fossa	Square	Doublet	200	NR	NR	NR	400	50	ITT
Phillips et al. (91)	Tibial nerve in popliteal fossa	Square	Singlet	100	-	NR	NR	NR	20	ITT
Place et al. (92)	Tibial nerve in popliteal fossa	Rectangular	Doublet	1000	10	40-100	NR	NR	50	ITT
Saldanha et al. (93)	Tibial nerve in popliteal fossa	Rectangular	Doublet	500	20	NR	NR	NR	NR	ITT
Sara et al. (a) (13)	Tibial nerve in popliteal fossa	Square	Doublet	100	NA	NR	100	400	10	ITT

Sara et al. (b) (21)	Tibial nerve in popliteal fossa	Square	Single	100	-	NR	NR	400	10	ITT
Scaglioni et al. (a) (94)	Belly of gastrocnemius muscle	Square	Single	50	-	NR	1	NR	NR	NR
Scaglioni et al. (b) (42)	Tibial nerve in popliteal fossa	Square	Single	1000	-	50-86	NR	400	NR	ITT
Scaglioni et al. (c) (95)	Tibial nerve in popliteal fossa	Square	Single	1000	-	50-86	NR	400	10	ITT
Shimoda et al. (96)	Tibial nerve in popliteal fossa	NR	Triplet	NR	10	NR	NR	NR	10-20	ITT
Siegler et al. (97)	Posterior tibial nerve in popliteal fossa	Square	Single	1000	-	70-95	NR	400	50	ITT
Simoneau et al. (98)	Tibial nerve in popliteal fossa	Rectangular	Doublet	1000	NR	NR	100	400	0	ITT
Stutzig et al. (99)	Tibial nerve in popliteal fossa	Rectangular	Doublet	1000	10	NR	NR	NR	20	ITT
Suzuki et al. (100)	Tibial nerve at the level of the popliteal fossa	NR	Doublet	200	NR	101.3(47.8)	NR	NR	10 mA	ITT
Trajano et al. (101)	Tibial nerve in popliteal fossa	Square	Single	1000	-	NR	NR	NR	20	ITT
Unhjem et al. (102)	Tibial nerve in popliteal fossa	Square	Single	1000	-	NR	NR	NR	50	ITT
Weir et al. (103)	Posterior tibial nerve	Square	Doublet	NR	10	NR	NR	NR	50	ITT

All data reported as mean (SD); NR, not reported; ITT, interpolated twitch technique; CAR, central activation ratio.

Supplementary file G. Individual study demographic information

Study	Group	Sample size	Sex (female)	Age	Height	Weight
Akagi et al. (a)	Healthy	12	0	22 (1)	170 (3.9)	64.7 (13.3)
Akagi et al. (b)	Young men	20	0	22 (2)	170.6 (5)	62.6 (6.5)
	Young women	20	20	22 (1)	157.4 (4.1)	51.7 (6.5)
	Older men	19	0	73 (5)	165.4 (6.5)	67.6 (10.3)
	Older women	14	14	72 (7)	154.5 (4.7)	56 (5.9)
Avela et al.	Healthy	8	0	25	NR	NR
Barber et al.	Older adults	16	7	70 (3)	166 (8)	73 (18)
	Younger adults	18	8	27 (3)	178 (9)	71 (13)
Behm et al.	Resistance trained	14	7	21.5 (4)	169.3 (6.3)	65.4 (5.2)
	Untrained controls	14	7	24.4 (5)	167.9 (6.3)	68.1 (7.1)
Cannavan et al.	Healthy	9	0	20.4 (0.7)	179.4 (6.3)	76 (8)
Cattagni et al. (a)	Non-fallers	23	17	83.3 (3.9)	159.9 (7.6)	61.8 (9.9)
	Fallers	25	18	84 (4.1)	161.2 (9.8)	63.4 (9.5)
Cattagni et al. (b)	Healthy	10	0	22.6 (4)	171.5 (11.9)	67.5 (13.4)
Crivelli et al.	Healthy	10	0	31 (5)	180 (5)	76 (6)
Crouzier et al.	Achilles tendinopathy	21	3	36.2 (8.3)	175.9 (8)	72.7 (8.7)
	Healthy	21	3	35.1 (7.7)	177.5 (7.8)	71.8 (10.5)
Cruz et al.	Healthy	10	0	27 (5)	176 (7)	79 (14)
Dalton et al. (a)	Older men	10	0	77.5 (3)	175.5 (7.9)	88.6 (13.1)
	Young men	10	0	24.1 (2.8)	175 (8.7)	78.6 (7.9)
Dalton et al. (b)	Knee flexion	11	0	26.3 (3.4)	178.1 (6.1)	80.1 (3.4)
	Knee extension	11	0	26.3 (3.4)	178.1 (6.1)	80.1 (3.4)
Dalton et al. (c)	Older men	6	0	75.3 (4.1)	177 (4.2)	88 (12.2)
	Young men	6	0	23.5 (2.9)	173.7 (8.5)	82.3 (11.8)
Dalton et al. (d)	Young men	10	0	26.3 (3.4)	178.1 (6.1)	80.1 (3.4)
	Older men	10	0	77.6 (4.4)	175.7 (3.8)	83.7 (11.3)
Ekblom et al.	Training group	9	4	27 (4.8)	180 (9)	73.8 (14.8)
	Control group	11	4	27 (6)	177 (10)	70.6 (10)
Ema et al.	Older men	17	0	74 (5)	164.3 (6.2)	67.6 (10.3)
	Older women	15	15	72 (4)	154.4 (4.6)	55.5 (6)
.	Young men	18	0	22 (2)	170.6 (5)	62.6 (6.5)
	Young women	19	19	22 (1)	157.1 (4)	50.9 (5.6)

Fimland et al.	Stroke	12	4	44.8 (36.5)	NR	NR
Girard et al.(a)	Healthy	17	0	27 (1)	184 (1)	80.1 (1.6)
Girard et al. (b)	Healthy	14	0	32 (4)	178 (5)	78 (8)
Gondin et al (a)	Intervention	8	0	25.8 (1.6)	176.4 (2)	70 (2.6)
	Control	9	0	24.7 (1.1)	181.3 (2.1)	76.8 (3.1)
Gondin et al. (b)	Intervention	12	0	21.7 (3.4)	176.6 (7.4)	70.5 (4.7)
	Control	7	0	26.6 (5.5)	178.3 (8.4)	71.9 (6.6)
Green et al.	Healthy	6	2	32	NR	NR
Grospretre et al.	Healthy	12	1	25.4 (8)	176 (6)	62.1 (7.5)
	Healthy	12	6	29.6 (10.5)	171 (7)	64.2 (9.2)
Guette et al.	Healthy	11	0	23.4 (0.7)	177.7 (2.1)	70 (2.2)
Hali et al.	Dorsiflexion	10	0	24 (3)	181 (5)	81 (7)
	Plantar flexion	10	0	24 (3)	181 (5)	81 (7)
Hartman et al.	Untrained	6	0	25.8 (4.9)	177.1 (8.7)	73.6 (8.7)
	Trained	8	0	25.7 (4.2)	175.9 (7.7)	78.1 (6.2)
Harwood et al.	Woman	8	8	24 (2)	164 (3)	58 (4)
	Men	8	0	24 (2)	187 (5)	96 (18)
Herda et al.	Woman	11	11	21 (2.5)	NR	52 (6)
	Men	14	0	21 (2.5)	NR	69 (9)
Hirata et al. (a)	Intervention 1	12	0	22 (1)	176 (8)	67 (6)
	Intervention 2	12	0	23 (1)	171 (6)	65 (9)
	Control	12	0	22 (1)	170 (4)	65 (13)
Hirata et al. (b)	Young adults	29	14	23 (3)	165.3 (8.4)	59.8 (11.9)
	Older adults	30	15	72 (5)	159.8 (9)	60.1 (11.9)
Hoffman et al.	Healthy	10	3	29.5 (4.6)	173.3 (7)	72.6 (10.6)
Hotta et al.	Healthy	11	0	21.6 (3.4)	170.2 (5.7)	60.8 (9.4)
Jo et al.	Men	10	0	27.3 (5.3)	183.5 (5.8)	91.5 (11.4)
	Woman	14	14	29.4 (4.9)	167.4 (7.3)	63.9 (9.8)
Kawakami et al.	Healthy	9	0	33.5	172 (4.2)	71.1 (9.7)
Kennedy et al.	Men	8	0	25	176.8 (4.9)	71.8 (6.7)
Kennedy et al.	Woman	6	6	25	170.5 (3.9)	60.5 (4.5)
Kirk et al.	Young men	10	0	27 (3)	180 (6)	81 (10)
	Older men	10	0	81 (4)	171 (7)	75 (14)
Klass et al.	Healthy	10	4	25	NR	NR

Klein et al.	Stroke	7	2	55.8 (3.6)	NR	NR
Knarr et al.	Stroke	17	2	60.7 (8.9)	NR	NR
Koryak et al. (a)	Healthy	6	0	38 (4.9)	179 (2.9)	79.5 (6.9)
Koryak et al. (b)	Healthy	4	4	31.5 (3.4)	162.3 (3.8)	55 (3.6)
Kubo et al. (a)	Intervention 1	10	0	22.3 (1.1)	171.4 (6.1)	63.8 (8.7)
	Intervention 2	10	0	22.5 (1.6)	169.8 (3.3)	62.7 (7.8)
Kubo et al. (b)	20-year olds	19	0	24.5 (2.1)	171.6 (3.9)	69.7 (10.3)
	30-year olds	15	0	35.2 (2.2)	171.5 (4.8)	72.4 (8.6)
	50-year olds	10	0	51.4 (4.4)	172.4 (4.3)	73.9 (8)
	70-year olds	15	0	69.7 (4.5)	159 (6.3)	59.4 (8)
Kuchinad et al.	Healthy	11	1	36	NR	NR
Lapole et al. (a)	Healthy	10	0	23.3 (5)	180 (7.5)	75.2 (9.6)
Lapole et al. (b)	Healthy	29	0	21.7 (1.7)	177 (9)	71.7 (14.1)
Maffiuletti et al.	Intervention	8	0	20.4 (2.1)	186.4 (8)	83.5 (9.6)
	Control	6	0	26 (5.1)	176.7 (4.8)	69.5 (4.6)
Marathamuthu et al.	Healthy	10	0	28.7 (4.24)	170 (4)	72.3 (6.53)
Merlet et al.	Healthy	12	5	22.5 (1.2)	172.5 (9.7)	63.5 (9.2)
Miller et al.	Healthy	14	0	22 (3)	177 (6)	84 (11)
Morse et al. (a)	Elderly	19	0	73.83 (3.5)	173.4 (4.4)	78.4 (8.3)
	Young	12	0	25.3 (4.4)	176.4 (7.7)	79.1 (11.9)
Morse et al. (b)	Young men	9	0	24.7 (4.7)	179.3 (7.8)	76.9 (12.4)
	Elderly men	9	0	83.7 (3.6)	170.4 (4.5)	77.5 (8.1)
Neyroud et al. (a)	Healthy	10	3	35 (10)	173 (6)	71 (10)
Neyroud et al. (b)	Healthy	14	3	27 (4)	175 (5)	72 (9)
Nordlund et al.	Healthy	10	0	28 (5)	179 (5)	79 (5)
Paris et al.	Healthy	13	2	24.2 (2.6)	NR	NR
Periard et al.	Healthy	12	0	22 (4)	183.5 (7.7)	80.8 (9.5)
Phillips et al.	Middle aged adults	22	22	54 (5.8)	165.6 (5.9)	72.2 (13)
	Young adults	17	17	21.8 (1.4)	166.8 (7.1)	70.4 (10.7)
Place et al.	Healthy	12	0	28 (4.8)	174.6 (6.3)	68.8 (7.5)
Saldanha et al.	Healthy	8	0	27.6 (2.4)	182 (8.7)	74.5 (0.7)
Sara et al. (a)	Men	14	0	21.5 (1.8)	181 (8)	79.4 (10.3)
	Woman	14	14	21.1 (2.9)	166 (7)	64 (10.8)
Sara et al. (b)	Control	14	7	23.6 (5.5)	148 (11)	67.4 (13)

	Achilles tendinopathy	14	4	31 (11.1)	152 (9)	87.7 (21.3)
Scaglioni et al. (a)	Healthy	14	0	68	172	80
Scaglioni et al. (b)	Healthy	11	0	26 (4)	178 (5)	72 (7)
Scaglioni et al. (c)	Young men	11	0	26 (4)	178 (5)	72 (7)
	Older men	13	0	76 (3)	168 (4)	73 (10)
Shimoda et al.	Intervention	10	0	22 (1.1)	170.9 (7.6)	64.8 (7.4)
	Control	10	0	21.9 (0.7)	174.2 (4.6)	65.4 (7.2)
Siegler et al.	Healthy	8	0	24 (2)	179.3 (6.2)	81.1 (11.9)
Simoneau et al.	Young adults	11	0	23.9 (1.7)	176 (4)	74 (8.53)
	Older adults	12	0	77.1 (1.8)	168 (3)	74.5 (10.9)
Stutzig et al.	Healthy	13	8	27.4 (5.1)	174.1 (8.2)	69.5 (12.6)
Suzuki et al.	Healthy	8	0	22 (3)	171.2 (6)	68.1 (10.3)
Trajano et al.	Healthy	13	0	26.5 (5)	172 (9)	71.1 (13.8)
Unhjem et al.	Sedentary older adults	10	0	71 (4)	179 (6)	94.7 (12.9)
	Recreationally active older adults	11	0	73 (6)	178 (8)	87 (17.6)
	Master older athletes	11	0	71 (4)	178 (9)	92.5 (12.9)
	Young adults	9	0	22 (2)	187 (5)	79.3 (12)
Weir et al.	Healthy	15	15	23.1 (2.1)	165.8 (5.8)	58.9 (3.8)

NR, Not reported; all data displayed as mean (SD)

Supplementary file H. Summary statistics for knee flexion

		Healthy					Achilles tendinopathy					Stroke				
		Valid N	Mean	Median	Minimum	Maximum	Valid N	Mean	Median	Minimum	Maximum	Valid N	Mean	Median	Minimum	Maximum
**0˚ knee flexion**	Sample size	65	13	12	6	25	2	18	18	14	21	2	15	15	12	17
	ITT (%)	63	89.11	90.70	52.00	100.00	2	89.55	89.55	81.20	97.90	1	29.00	29.00	29.00	29.00
	CAR	10	78.84	99.05	.67	100.00	0					1	.39	.39	.39	.39
	MVC (Nm)	54	116.21	120.00	34.70	229.00	2	128.6	128.60	121.10	136.10	2	51.95	51.95	33.90	70.00
**5˚ knee flexion**	Sample size	1	10	10	10	10	0					0				
	ITT (%)	1	97.12	97.12	97.12	97.12	0					0				
	CAR	0					0					0				
	MVC (Nm)	0					0					0				
**10˚ knee flexion**	Sample size	3	10	10	10	11	0					0				
	ITT (%)	3	96.10	96.30	95.00	97.00	0					0				
	CAR	0					0					0				
	MVC (Nm)	3	183.33	192.30	154.20	203.50	0					0				
**20˚ knee flexion**	Sample size	2	10	10	10	10	0					0				

	ITT (%)	2	94.38	94.38	94.10	94.65	0					0				
	CAR	0					0					0				
	MVC	2	146.63	146.63	125.85	167.40	0					0				
**30˚ knee flexion**	Sample size	3	17	17	12	22	0					0				
	ITT (%)	3	91.57	92.70	88.80	93.20	0					0				
	CAR	0					0					0				
	MVC (Nm)	3	128.80	136.40	109.00	141.00	0					0				
**60˚ knee flexion**	Sample size	6	13	10	8	29	0					0				
	ITT (%)	6	88.95	94.85	52.80	99.10	0					0				
	CAR	2	98.25	98.25	98.10	98.40	0					0				
	MVC (Nm)	6	144.43	135.90	117.00	214.80	0					0				
**80˚ knee flexion**	Sample size	1	13	13	13	13	0					0				
	ITT (%)	1	89.80	89.80	89.80	89.80	0					0				
	CAR	0					0					0				
	MVC (Nm)	1	154.10	154.10	154.10	154.10	0					0				
**90˚ knee flexion**	Sample size	47	11	11	4	19	0					1	7	7	7	7
	ITT (%)	47	92.49	95.60	62.40	99.50	0					1	48.50	48.50	48.50	48.50

	CAR	0					0					0				
	MVC (Nm)	39	143.99	141.00	85.20	284.80	0					1	56.70	56.70	56.70	56.70
**100˚ knee flexion**	Sample size	1	12	12	12	12	0					0				
	ITT (%)	1	95.90	95.90	95.90	95.90	0					0				
	CAR	0					0					0				
	MVC (Nm)	1	82.70	82.70	82.70	82.70	0					0				

ITT, interpolated twitch technique; CAR, central activation ratio; MVC, maximal voluntary contraction; Nm, newton meters

Supplementary file I. Methodological quality of included studies

Author	Were the criteria for inclusion in the sample clearly defined?	Were the study participants and the setting described in detail?	Was the exposure measured in a valid and reliable way?	Were objective, standard criteria used for measurement of the condition?	Were confounding factors identified?	Were strategies to deal with confounding factors stated?	Were the outcomes measured in a valid and reliable way?	Was appropriate statistical analysis used?	Quality
Akagi et al. (a)	n	y	u		n	n	y	y	Low
Akagi et al. (b)	n	y	y		n	y	y	y	Low
Avela et al.	n	n	y		n	n	n	y	Low
Barber et al.	n	y	y		n	y	y	y	Low
Behm et al.	n	y	n		n	n	n	y	Low
Cannavan et al.	n	y	y		n	n	y	y	Low
Cattagni et al. (a)	y	y	y		y	n	y	y	Low
Cattagni et al. (b)	n	y	y		n	n	y	n	Low
Crivelli et al.	n	y	u		n	n	y	y	Low
Crouzier et al.	y	y	y	y	n	n	y	y	Low
Cruz et al.	y	y	n		y	n	y	n	Low
Dalton et al. (a)	y	y	u		y	n	y	y	Low
Dalton et al. (b)	n	y	u		n	n	n	y	Low
Dalton et al. (c)	y	y	u		n	n	n	y	Low
Dalton et al. (d)	y	y	y		y	n	n	y	Low
Ekblom et al.	n	y	y		n	n	y	y	Low
Ema et al.	n	y	y		n	n	n	y	Low

Fimland et al.	y	n	y	y	y	n	y	y	Low
Girard et al. (a)	y	y	y		y	y	y	y	High
Girard et al. (b)	u	y	y		n	n	y	y	Low
Gondin et al. (a)	y	y	n		y	n	y	y	Low
Gondin et al. (b)	n	y	y		n	n	y	y	Low
Green et al.	y	n	y		n	n	n	n	Low
Grospretre et al.	y	y	y		y	n	y	y	Low
Guette et al.	n	y	n		n	n	y	y	Low
Hali et al.	y	y	y		y	n	y	n	Low
Hartman et al.	y	y	y		y	n	y	y	Low
Harwood et al.	y	y	n		y	n	y	y	Low
Herda et al.	y	n	y		y	n	n	y	Low
Hirata et al. (a)	y	y	y		y	n	y	y	Low
Hirata et al. (b)	y	y	y		y	y	y	y	High
Hoffman et al.	y	y	y		n	n	y	y	Low
Hotta et al.	n	y	y		n	n	n	y	Low
Jo et al.	y	y	u		y	y	y	y	Low
Kawakami et al.	n	y	n		n	n	u	y	Low
Kennedy et al.	n	y	n		n	n	y	y	Low
Kirk et al.	y	y	u		n	n	y	y	Low
Klass et al.	n	n	y		n	n	y	n	Low
Klein et al.	y	y	y	y	y	n	y	y	Low
Knarr et al.	n	n	n	n	n	n	n	y	Low
Koryak et al. (a)	n	y	n		n	n	n	n	Low
Koryak et al. (b)	n	y	n		n	n	n	n	Low
Kubo et al. (a)	n	y	n		n	n	n	y	Low
Kubo et al. (b)	n	y	n		n	n	n	y	Low

Kuchinad et al.	y	n	y		y	n	y	y	Low
Lapole et al. (a)	u	y	y		n	n	y	y	Low
Lapole et al. (b)	n	y	y		n	n	n	y	Low
Maffiuletti et al.	u	y	u		y	n	y	y	Low
Marathamuthu et al.	y	y	y		y	n	y	y	Low
Merlet et al.	y	y	y		y	n	y	y	Low
Miller et al.	y	y	y		y	n	y	y	Low
Morse et al. (a)	y	y	u		y	n	y	n	Low
Morse et al. (b)	y	y	n		y	n	n	y	Low
Neyroud et al. (a)	n	y	y		n	n	y	y	Low
Neyroud et al. (b)	n	y	y		n	n	y	y	Low
Nordlund et al.	n	y	y		n	n	y	y	Low
Paris et al.	n	n	y		n	n	n	y	Low
Periard et al.	n	y	y		n	n	y	y	Low
Phillips et al.	y	y	y		y	n	y	y	Low
Place et al.	u	y	y		n	n	y	y	Low
Saldanha et al.	n	y	n		n	n	n	y	Low
Sara et al. (a)	y	y	u		y	y	n	y	Low
Sara et al. (b)	y	y	u	y	y	y	u	y	Low
Scaglioni et al. (a)	y	y	n		n	n	n	y	Low
Scaglioni et al. (b)	y	y	n		y	n	y	y	Low
Scaglioni et al. (c)	y	y	u		y	n	y	y	Low
Shimoda et al.	y	y	u		y	n	n	y	Low
Siegler et al.	n	y	y		n	n	n	y	Low
Simoneau et al.	y	y	n		y	n	y	y	Low
Stutzig et al.	y	y	y		y	n	y	y	Low

Suzuki et al.	y	y	u		n	n	y	y	Low
Trajano et al.	y	y	y		y	n	n	y	Low
Unhjem et al.	y	y	y		n	n	y	y	Low
Weir et al.	y	y	n		n	n	y	y	Low

Supplementary file J. Egger's Regression-Based Test[a,b]

	Parameter	Coefficient	Std. Error	t	Sig. (2-tailed)	95% Confidence Interval	
						Lower	Upper
**Healthy**	Intercept	101.208	1.4917	67.847	<.001	98.254	104.161
	Standard error[c]	-3.870	0.4283	-9.034	<.001	-4.718	-3.022
	Age	-0.062	0.0261	-2.386	0.019	-0.114	-0.011
	Knee position	0.004	0.0126	0.329	0.743	-0.021	0.029
	Ankle position	-0.072	0.0762	-0.949	0.345	-0.223	0.079
**Overall**	Intercept	101.817	1.4665	69.428	<0.001	98.914	104.719
	Standard error[c]	-4.180	0.3919	-10.668	<.001	-4.956	-3.405
	Age	-0.064	0.0267	-2.400	0.018	-0.117	-0.011
	Knee position	0.004	0.0127	0.284	0.777	-0.022	0.029
	Ankle position	-0.069	0.0777	-0.894	0.373	-0.223	0.084

a. Random-effects meta-regression
b. Regression Based Test cannot be computed for subgroup(s) Population = Achilles tendinopathy, Stroke.
c. Standard error of effect size

Supplementary file L. GRADE assessment per sub-group

Subgroup	Risk of bias	Small sample bias	Inconsistency	Imprecision	Indirectness	Publication bias	Certainty
**Healthy populations**	Downgrade	Do not downgrade (n=1,629)	Downgrade (I^2 = 100%).	Do not downgrade (95% CI=89.8 to 92.6)	N/A	Downgrade (Egger's regression = -3.87, p<0.001)	Very low
**Achilles tendinopathy**	Downgrade	Downgrade (n=35)	Downgrade (I^2 = 89%)	Downgrade (95% CI=74.1 to 106.7)	N/A	N/A	Very low
**Stroke**	Downgrade	Downgrade (n=36)	Do not downgrade (I^2 = 32%)	Downgrade (95% CI=17.4 to 53.3)	N/A	N/A	Very low

The certainty of the evidence was downgraded for risk of bias if >50% of studies were deemed as high risk of bias by the assessment of methodological quality criteria. The certainty of the evidence was downgraded for small sample bias when studies had <200 participants. The certainty of the evidence was downgraded for inconsistency if statistical heterogeneity ($P<0.10$ or $I^2 \geq 40\%$). The certainty of the evidence was downgraded for imprecision if wide confidence intervals or confidence intervals which crossed zero were present. The certainty of the evidence was downgraded for publication bias if the standard error coefficient in Egger's test was significantly different from zero. Indirectness was not judged in this review as it was not relevant.

Supplementary file references

1. Akagi R, Imaizumi N, Sato S, Hirata N, Tanimoto H, Hirata K. Active recovery has a positive and acute effect on recovery from fatigue induced by repeated maximal voluntary contractions of the plantar flexors. J Electromyogr Kinesiol. 2020;50:102384.
2. Akagi R, Suzuki M, Kawaguchi E, Miyamoto N, Yamada Y, Ema R. Muscle size-strength relationship including ultrasonographic echo intensity and voluntary activation level of a muscle group. Arch Gerontol Geriatr. 2018;75:185-90.
3. Avela J, Finni T, Liikavainio T, Niemelä E, Komi PV. Neural and mechanical responses of the triceps surae muscle group after 1 h of repeated fast passive stretches. J Appl Physiol (1985). 2004;96(6):2325-32.
4. Barber LA, Barrett RS, Gillett JG, Cresswell AG, Lichtwark GA. Neuromechanical properties of the triceps surae in young and older adults. Exp Gerontol. 2013;48(11):1147-55.
5. Behm D, St-Pierre D. Fatigue Mechanisms in Trained and Untrained Plantar Flexors. The Journal of Strength & Conditioning Research. 1998;12.
6. Cannavan D, Coleman DR, Blazevich AJ. Lack of effect of moderate-duration static stretching on plantar flexor force production and series compliance. Clin Biomech (Bristol). 2012;27(3):306-12.
7. Cattagni T, Harnie J, Jubeau M, Hucteau E, Couturier C, Mignardot JB, et al. Neural and muscular factors both contribute to plantar-flexor muscle weakness in older fallers. Exp Gerontol. 2018;112:127-34.
8. Cattagni T, Billet C, Cornu C, Jubeau M. No Alteration of the Neuromuscular Performance of Plantar-Flexor Muscles After Achilles Tendon Vibration. J Sport Rehabil. 2017;26(2):1-3.
9. Crivelli G, Borrani F, Capt R, Gremion G, Maffiuletti NA. Actions of β2-adrenoceptor agonist drug on human soleus muscle contraction. Med Sci Sports Exerc. 2013;45(7):1252-60.
10. Crouzier M, Tucker K, Lacourpaille L, Doguet V, Fayet G, Dauty M, et al. Force-sharing within the Triceps Surae: An Achilles Heel in Achilles Tendinopathy. Med Sci Sports Exerc. 2020;52(5):1076-87.
11. Cruz R, Tramontin AF, Oliveira AS, Caputo F, Denadai BS, Greco CC. Ischemic preconditioning increases spinal excitability and voluntary activation during maximal plantar flexion contractions in men. Scand J Med Sci Sports. 2024;34(3):e14591.
12. Dalton BH, Power GA, Vandervoort AA, Rice CL. Power loss is greater in old men than young men during fast plantar flexion contractions. J Appl Physiol (1985). 2010;109(5):1441-7.
13. Dalton BH, Power GA, Allen MD, Vandervoort AA, Rice CL. The genu effect on plantar flexor power. Eur J Appl Physiol. 2013;113(6):1431-9.
14. Dalton BH, Harwood B, Davidson AW, Rice CL. Recovery of motoneuron output is delayed in old men following high-intensity fatigue. J Neurophysiol. 2010;103(2):977-85.
15. Dalton BH, Allen MD, Power GA, Vandervoort AA, Rice CL. The effect of knee joint angle on plantar flexor power in young and old men. Exp Gerontol. 2014;52:70-6.
16. Ekblom MM. Improvements in dynamic plantar flexor strength after resistance training are associated with increased voluntary activation and V-to-M ratio. J Appl Physiol (1985). 2010;109(1):19-26.
17. Ema R, Kawaguchi E, Suzuki M, Akagi R. Plantar flexor strength at different knee positions in older and young males and females. Exp Gerontol. 2020;142:111148.
18. Fimland MS, Moen PM, Hill T, Gjellesvik TI, Tørhaug T, Helgerud J, et al. Neuromuscular performance of paretic versus non-paretic plantar flexors after stroke. Eur J Appl Physiol. 2011;111(12):3041-9.
19. Girard O, Nybo L, Mohr M, Racinais S. Plantar flexor neuromuscular adjustments following match-play football in hot and cool conditions. Scand J Med Sci Sports. 2015;25 Suppl 1:154-63.
20. Girard O, Banzet S, Koulmann N, Chennaoui M, Drogou C, Chalabi H, et al. Larger strength losses and muscle activation deficits in plantar flexors induced by backward downhill in reference to distance-matched forward uphill treadmill walk. Eur J Sport Sci. 2018;18(10):1346-56.
21. Gondin J, Guette M, Maffiuletti NA, Martin A. Neural activation of the triceps surae is impaired following 2 weeks of immobilization. Eur J Appl Physiol. 2004;93(3):359-65.
22. Gondin J, Duclay J, Martin A. Soleus- and gastrocnemii-evoked V-wave responses increase after neuromuscular electrical stimulation training. J Neurophysiol. 2006;95(6):3328-35.
23. Green S, Robinson E, Wallis E. Assessment of calf muscle fatigue during submaximal exercise using transcranial magnetic stimulation versus transcutaneous motor nerve stimulation. Eur J Appl Physiol. 2014;114(1):113-21.
24. Grosprêtre S, Gimenez P, Thouvenot A, Patoz A, Lussiana T, Mourot L. Different plantar flexors neuromuscular and mechanical characteristics depending on the preferred running form. J Electromyogr Kinesiol. 2021;59:102568.

25. Guette M, Gondin J, Martin A, Pérot C, Van Hoecke J. Plantar flexion torque as a function of time of day. Int J Sports Med. 2006;27(3):171-7.
26. Hali K, Zero AM, Rice CL. Effect of ankle joint position on triceps surae contractile properties and motor unit discharge rates. Physiol Rep. 2021;8(24):e14680.
27. Hartman MJ, Ryan ED, Cramer JT, Bemben MG. The effects of fatigue of the plantar flexors on peak torque and voluntary activation in untrained and resistance-trained men. J Strength Cond Res. 2011;25(2):527-32.
28. Harwood B, Scherer J, Brown RE, Cornett KMD, Kenno KA, Jakobi JM. Neuromuscular responses of the plantar flexors to whole-body vibration. Scand J Med Sci Sports. 2017;27(12):1569-75.
29. Herda TJ, Walter AA, Costa PB, Ryan ED, Stout JR, Cramer JT. Differences in the log-transformed electromyographic-force relationships of the plantar flexors between high- and moderate-activated subjects. J Electromyogr Kinesiol. 2011;21(5):841-6.
30. Hirata K, Tanimoto H, Sato S, Hirata N, Imaizumi N, Sugihara Y, et al. Carbon dioxide hydrate as a recovery tool after fatigue of the plantar flexors. J Biomech. 2020;108:109900.
31. Hirata K, Iida N, Kanda A, Shoji M, Yoshida T, Yamada Y, et al. Association of age-related decrease in intracellular-to-total water ratio with that in explosive strength of the plantar flexors: a cross-sectional study. J Physiol Anthropol. 2022;41(1):10.
32. Hoffman BW, Oya T, Carroll TJ, Cresswell AG. Increases in corticospinal responsiveness during a sustained submaximal plantar flexion. J Appl Physiol (1985). 2009;107(1):112-20.
33. Hotta N, Ishida K, Sato K, Koike T, Katayama K, Akima H. The effect of intense interval cycle-training on unloading-induced dysfunction and atrophy in the human calf muscle. J Physiol Anthropol. 2011;30(1):29-35.
34. Jo D, Goubran M, Bilodeau M. Sex differences in central and peripheral fatigue induced by sustained isometric ankle plantar flexion. J Electromyogr Kinesiol. 2022;65:102676.
35. Kawakami Y, Amemiya K, Kanehisa H, Ikegawa S, Fukunaga T. Fatigue responses of human triceps surae muscles during repetitive maximal isometric contractions. J Appl Physiol (1985). 2000;88(6):1969-75.
36. Kennedy A, Hug F, Sveistrup H, Guével A. Fatiguing handgrip exercise alters maximal force-generating capacity of plantar-flexors. Eur J Appl Physiol. 2013;113(3):559-66.
37. Kirk EA, Copithorne DB, Dalton BH, Rice CL. Motor unit firing rates of the gastrocnemii during maximal and sub-maximal isometric contractions in young and old men. Neuroscience. 2016;330:376-85.
38. Klass M, Guissard N, Duchateau J. Limiting mechanisms of force production after repetitive dynamic contractions in human triceps surae. J Appl Physiol (1985). 2004;96(4):1516-21; discussion.
39. Klein CS, Brooks D, Richardson D, McIlroy WE, Bayley MT. Voluntary activation failure contributes more to plantar flexor weakness than antagonist coactivation and muscle atrophy in chronic stroke survivors. J Appl Physiol (1985). 2010;109(5):1337-46.
40. Knarr BA, Ramsay JW, Buchanan TS, Higginson JS, Binder-Macleod SA. Muscle volume as a predictor of maximum force generating ability in the plantar flexors post-stroke. Muscle Nerve. 2013;48(6):971-6.
41. Koryak Y. Contractile properties of the human triceps surae muscle during simulated weightlessness. Eur J Appl Physiol Occup Physiol. 1995;70(4):344-50.
42. Koryak YA. Influence of simulated microgravity on mechanical properties in the human triceps surae muscle in vivo. I: effect of 120 days of bed-rest without physical training on human muscle musculo-tendinous stiffness and contractile properties in young women. Eur J Appl Physiol. 2014;114(5):1025-36.
43. Kubo K, Ikebukuro T, Yata H, Tsunoda N, Kanehisa H. Effects of training on muscle and tendon in knee extensors and plantar flexors in vivo. J Appl Biomech. 2010;26(3):316-23.
44. Kubo K, Morimoto M, Komuro T, Tsunoda N, Kanehisa H, Fukunaga T. Age-related differences in the properties of the plantar flexor muscles and tendons. Med Sci Sports Exerc. 2007;39(3):541-7.
45. Kuchinad RA, Ivanova TD, Garland SJ. Modulation of motor unit discharge rate and H-reflex amplitude during submaximal fatigue of the human soleus muscle. Exp Brain Res. 2004;158(3):345-55.
46. Lapole T, Ahmaidi S, Gaillien B, Leprêtre PM. Influence of dorsiflexion shoes on neuromuscular fatigue of the plantar flexors after combined tapping-jumping exercises in volleyball players. J Strength Cond Res. 2013;27(7):2025-33.
47. Lapole T, Pérot C. Effects of repeated Achilles tendon vibration on triceps surae force production. J Electromyogr Kinesiol. 2010;20(4):648-54.
48. Maffiuletti NA, Pensini M, Martin A. Activation of human plantar flexor muscles increases after electromyostimulation training. J Appl Physiol (1985). 2002;92(4):1383-92.
49. Marathamuthu S, Selvanayagam VS, Yusof A. Contralateral Effects of Eccentric Exercise and DOMS of the Plantar Flexors: Evidence of Central Involvement. Res Q Exerc Sport. 2022;93(2):240-9.

50. Merlet AN, Cattagni T, Cornu C, Jubeau M. Effect of knee angle on neuromuscular assessment of plantar flexor muscles: A reliability study. PLoS One. 2018;13(3):e0195220.
51. Miller JD, Herda TJ, Trevino MA, Mosier EM. The effects of passive stretching plus vibration on strength and activation of the plantar flexors. Appl Physiol Nutr Metab. 2016;41(9):917-23.
52. Morse CI, Thom JM, Reeves ND, Birch KM, Narici MV. In vivo physiological cross-sectional area and specific force are reduced in the gastrocnemius of elderly men. J Appl Physiol (1985). 2005;99(3):1050-5.
53. Morse CI, Thom JM, Birch KM, Narici MV. Tendon elongation influences the amplitude of interpolated doublets in the assessment of activation in elderly men. J Appl Physiol (1985). 2005;98(1):221-6.
54. Neyroud D, Temesi J, Millet GY, Verges S, Maffiuletti NA, Kayser B, et al. Comparison of electrical nerve stimulation, electrical muscle stimulation and magnetic nerve stimulation to assess the neuromuscular function of the plantar flexor muscles. Eur J Appl Physiol. 2015;115(7):1429-39.
55. Neyroud D, Dodd D, Gondin J, Maffiuletti NA, Kayser B, Place N. Wide-pulse-high-frequency neuromuscular stimulation of triceps surae induces greater muscle fatigue compared with conventional stimulation. J Appl Physiol (1985). 2014;116(10):1281-9.
56. Nordlund MM, Thorstensson A, Cresswell AG. Central and peripheral contributions to fatigue in relation to level of activation during repeated maximal voluntary isometric plantar flexions. J Appl Physiol (1985). 2004;96(1):218-25.
57. Paris MT, Kulkarni SV, Rice CL. Electrically Evoked Isotonic Plantar Flexion Contractions Are Impaired Less than Voluntary After a Dynamic Fatiguing Task. Med Sci Sports Exerc. 2023;55(11):2096-102.
58. Périard JD, Girard O, Racinais S. Neuromuscular adjustments of the knee extensors and plantar flexors following match-play tennis in the heat. British Journal of Sports Medicine. 2014;48(Suppl 1):i45-i51.
59. Phillips KC, Noh B, Gage M, Yoon T. Neural and muscular alterations of the plantar flexors in middle-aged women. Exp Gerontol. 2022;159:111674.
60. Place N, Duclay J, Lepers R, Martin A. Unchanged H-reflex during a sustained isometric submaximal plantar flexion performed with an EMG biofeedback. J Electromyogr Kinesiol. 2009;19(6):e395-402.
61. Saldanha A, Nordlund Ekblom MM, Thorstensson A. Central fatigue affects plantar flexor strength after prolonged running. Scand J Med Sci Sports. 2008;18(3):383-8.
62. Sara L, Gutsch S, Hunter S. The single-leg heel raise does not predict maximal plantar flexion strength in healthy males and females. PLOS ONE. 2021;16:e0253276.
63. Sara LK, Gutsch SB, Bement MH, Hunter SK. Plantar Flexor Weakness and Pain Sensitivity Cannot Be Assumed in Midportion Achilles Tendinopathy. Exerc Sport Mov. 2023;1(4):1-7.
64. Scaglioni G, Ferri A, Minetti AE, Martin A, Van Hoecke J, Capodaglio P, et al. Plantar flexor activation capacity and H reflex in older adults: adaptations to strength training. J Appl Physiol (1985). 2002;92(6):2292-302.
65. Scaglioni G, Martin A. Assessment of plantar flexors activation capacity: nerve versus muscle stimulation by single versus double pulse. Eur J Appl Physiol. 2009;106(4):563-72.
66. Scaglioni G, Narici MV, Martin A. Neural Activation During Submaximal Contractions Seems More Reflective of Neuromuscular Ageing than Maximal Voluntary Activation. Front Aging Neurosci. 2016;8:19.
67. Shimoda M, Enomoto M, Horie M, Miyakawa S, Yagishita K. Effects of hyperbaric oxygen on muscle fatigue after maximal intermittent plantar flexion exercise. J Strength Cond Res. 2015;29(6):1648-56.
68. Siegler JC, Marshall P, Pouslen MK, Nielsen NP, Kennedy D, Green S. The effect of pH on fatigue during submaximal isometric contractions of the human calf muscle. Eur J Appl Physiol. 2015;115(3):565-77.
69. Simoneau E, Martin A, Van Hoecke J. Muscular performances at the ankle joint in young and elderly men. J Gerontol A Biol Sci Med Sci. 2005;60(4):439-47.
70. Stutzig N, Siebert T. Reproducibility of electromyographic and mechanical parameters of the triceps surae during submaximal and maximal plantar flexions. Muscle Nerve. 2016;53(3):464-70.
71. Suzuki T, Shioda K, Kinugasa R, Fukashiro S. Simultaneous Knee Extensor Muscle Action Induces an Increase in Voluntary Force Generation of Plantar Flexor Muscles. J Strength Cond Res. 2017;31(2):365-71.
72. Trajano GS, Seitz L, Nosaka K, Blazevich AJ. Contribution of central vs. peripheral factors to the force loss induced by passive stretch of the human plantar flexors. J Appl Physiol (1985). 2013;115(2):212-8.
73. Unhjem R, Nygård M, van den Hoven LT, Sidhu SK, Hoff J, Wang E. Lifelong strength training mitigates the age-related decline in efferent drive. J Appl Physiol (1985). 2016;121(2):415-23.
74. Weir DE, Tingley J, Elder GC. Acute passive stretching alters the mechanical properties of human plantar flexors and the optimal angle for maximal voluntary contraction. Eur J Appl Physiol. 2005;93(5-6):614-23.
