# Supplementary material for: Voluntary activation of the ankle plantar flexors: A systematic review and meta-analysis
Source: JSAMS Plus. 2025 Aug 29;6:100117. doi: 10.1016/j.jsampl.2025.100117 (PMC13008425; doi:10.1016/j.jsampl.2025.100117)
Supplement: Multimedia component 2 [file mmc2.docx]

**Supplementary file A. Search strategy**

| **Number** | **Combiners** | **Region** | **Terms** |
| --- | --- | --- | --- |
| 1 | Problem of Interest | Title | Calf OR Gastrocnemius OR Soleus OR triceps surae OR Achilles OR Plantar flex* |
| 2 | Outcome | All | Voluntary activation OR Interpolated Twitch OR Superimposed twitch* OR Resting twitch* OR Central activation* |
| 3 |  |  | #1 AND #2 |
|  | Limitations |  | Human |

**Supplementary file B. Search Strategy Documentation**

| **Source:** | **Date of search** | **Search strategy used (keywords & Boolean)** | **Search Limits (e.g., dates, language)** | **# results found** | **Comments** |
| --- | --- | --- | --- | --- | --- |
| PUBMED | 14/03/2024 | (Calf[Title] OR Gastrocnemius[Title] OR Soleus[Title] OR triceps surae[Title] OR Achilles[Title] OR Plantar flex*[Title]) AND (Voluntary activation OR Interpolated Twitch OR Superimposed twitch* OR Resting twitch* OR Central activation*) | Humans | 578 | Exported to End Note |
| CINAHL (Full Text) | 14/03/2024 | TI ( Calf OR Gastrocnemius OR Soleus OR triceps surae OR Achilles OR Plantar flex* ) AND TX ( Voluntary activation OR Interpolated Twitch OR Superimposed twitch* OR Resting twitch* OR Central activation* ) | Humans | 34 | Exported to End Note |
| EBSCO (Medline) | 14/03/2024 | TI ( Calf OR Gastrocnemius OR Soleus OR triceps surae OR Achilles OR Plantar flex* ) AND TX ( Voluntary activation OR Interpolated Twitch OR Superimposed twitch* OR Resting twitch* OR Central activation* ) | Humans | 104 | Exported to End Note |
| Cochrane Library (including clinicaltrials.gov and International Clinical Trials Registry Platform) | 14/03/2024 | Calf OR Gastrocnemius OR Soleus OR triceps surae OR Achilles OR Plantar flex* in Record Title AND Voluntary activation OR Interpolated Twitch OR Superimposed twitch* OR Resting twitch* OR Central activation* in All Text - (Word variations have been searched) |  | 106 | Exported to End Note |
| SportsDISCUS | 14/03/2024 | TI ( Calf OR Gastrocnemius OR Soleus OR triceps surae OR Achilles OR Plantar flex* ) AND TX ( Voluntary activation OR Interpolated Twitch OR Superimposed twitch* OR Resting twitch* OR Central activation* ) |  | 110 | Exported to End Note |
| Web of Science | 14/03/2024 | TI ( Calf OR Gastrocnemius OR Soleus OR triceps surae OR Achilles OR Plantar flex* ) AND TX ( Voluntary activation OR Interpolated Twitch OR Superimposed twitch* OR Resting twitch* OR Central activation* ) |  | 956 | Exported to End Note |
| Proquest | 14/03/2024 | title(Calf OR Gastrocnemius OR Soleus OR triceps surae OR Achilles OR Plantar flex*) AND (Voluntary activation OR Interpolated Twitch OR Superimposed twitch* OR Resting twitch* OR Central activation*) |  | 142 | Exported to End Note |
| TOTAL | | | | | 2030 |

**Supplementary file C. Quality assessment criteria**

| **Item Number** | **Item Definition** | **Criteria for a judgement of ‘YES’** |
| --- | --- | --- |
| 1 | Were the criteria for inclusion in the sample clearly defined? | Clear study inclusion criteria were defined, with sufficient detail to allow replication of the study. Study screened for musculoskeletal and neurological injury history. |
| 2 | Were the study participants and the setting described in detail? | Study subject characteristics (age, sex, body mass index, medical co-morbidities) and setting information were clearly defined and included sufficient detail to allow replication of the study. |
| 3 | Was the exposure measured in a valid and reliable way? | The study reported sufficient detail surrounding electrical stimulation protocol and used supramaximal stimulation of >120%. |
| 4 | Were objective, standard criteria used for measurement of the condition? | Where relevant, criteria used to measure the presence of pathology was clearly defined and used objective, standard criteria. |
| 5 | Were confounding factors identified? | Confounding factors, which may influence study results are identified in the study design. |
| 6 | Were strategies to deal with confounding factors stated? | Appropriate analysis employed to attempt to account for identified confounding factors. |
| 7 | Were the outcomes measured in a valid and reliable way? | Methods for voluntary activation and maximal isometric strength assessment were clearly described and were measured in a valid and reliable way. |
| 8 | Was appropriate statistical analysis used? | The statistical model used within the study was appropriate for their research question and a valid voluntary activation formula was used. |

**Supplementary file D. Individual study information**

| Study | Country | Study design | Population |
| --- | --- | --- | --- |
| Akagi et al. (a) (52) | Japan | Cross over | Healthy |
| Akagi et al. (b) (38) | Japan | Cross-sectional | Healthy |
| Avela et al. (53) | Finland | Quasi-experimental | Healthy |
| Barber et al. (54) | Australia | Cross-sectional | Healthy |
| Behm et al. (55) | Canada | Quasi-experimental | Healthy |
| Cannavan et al. (56) | England | Cross over | Healthy |
| Cattagni et al. (a) (57) | France | Cross-sectional | Healthy |
| Cattagni et al. (b) (58) | France | Quasi-experimental | Healthy |
| Crivelli et al. (59) | Switzerland | Cross over | Healthy |
| Crouzier et la. (32) | France | Cross-sectional | Achilles tendinopathy |
| Cruz et al. (60) | Brazil | Quasi-experimental | Healthy |
| Dalton et al. (a) (61) | Canada | Cross-sectional | Healthy |
| Dalton et al. (b) (17) | Canada | Cross-sectional | Healthy |
| Dalton et al. (c) (62) | Canada | Cross-sectional | Healthy |
| Dalton et al. (d) (16) | Canada | Cross-sectional | Healthy |
| Ekblom et al. (63) | Sweden | Randomised control trial | Healthy |
| Ema et al. (18) | Japan | Cross-sectional | Healthy |
| Fimland et al. (36) | Norway | Cross-sectional | Stroke |
| Girard et al. (a) (64) | France | Cross over | Healthy |
| Girard et al. (b) (65) | Switzerland | Quasi-experimental | Healthy |
| Gondin et al. (a) (66) | France | Randomised control trial | Healthy |
| Gondin et al. (b) (67) | France | Randomised control trial | Healthy |
| Green et al. (68) | New Zealand | Cross-sectional | Healthy |
| Grospretre et al. (69) | France | Cross-sectional | Healthy |
| Guette et al. (70) | France | Cross-sectional | Healthy |
| Hali et al. (19) | Canada | Cross over | Healthy |
| Hartman et al. (71) | USA | Cross-sectional | Healthy |
| Harwood et al. (72) | Canada | Quasi-experimental | Healthy |
| Herda et al. (73) | USA | Cross-sectional | Healthy |
| Hirata et al. (a) (74) | Japan | Randomised control trial | Healthy |
| Hirata et al. (b) (75) | Japan | Cross-sectional | Healthy |
| Hoffman et al. (76) | Australia | Quasi-experimental | Healthy |
| Hotta et al. (77) | Japan | Quasi-experimental | Healthy |
| Jo et al. (78) | Canada | Cross-sectional | Healthy |
| Kawakami et al. (79) | Japan | Cross over | Healthy |
| Kennedy et al. (80) | Canada | Quasi-experimental | Healthy |
| Kirk et al. (81) | Canada | Cross-sectional | Healthy |
| Klass et al. (82) | Belgium | Quasi-experimental | Healthy |
| Klein et al. (35) | Canada | Cross-sectional | Stroke |
| Knarr et al. (34) | USA | Cross-sectional | Stroke |
| Koryak et al. (a) (83) | Russia | Quasi-experimental | Healthy |
| Koryak et al. (b) (37) | Russia | Quasi-experimental | Healthy |
| Kubo et al. (a) (84) | Japan | Randomised control trial | Healthy |
| Kubo et al. (b) (85) | Japan | Cross-sectional | Healthy |
| Kuchinad et al. (86) | Canada | Quasi-experimental | Healthy |
| Lapole et al. (a) (87) | France | Cross over | Healthy |
| Lapole et al. (b) (88) | France | Quasi-experimental | Healthy |
| Maffiuletti et al. (89) | France | Quasi-Experimental | Healthy |
| Marathamuthu et al. (90) | Malaysia | Cross over | Healthy |
| Merlet et al. (20) | France | Reliability | Healthy |
| Miller et al. (91) | USA | Cross over | Healthy |
| Morse et al. (a) (92) | England | Cross-sectional | Healthy |
| Morse et al. (b) (21) | England | Cross-sectional | Healthy |
| Neyroud et al. (a) (93) | Switzerland | Cross-sectional | Healthy |
| Neyroud et al. (b) (94) | Switzerland | Quasi-experimental | Healthy |
| Nordlund et al. (95) | Sweden | Quasi-experimental | Healthy |
| Paris et al. (96) | Canada | Quasi-experimental | Healthy |
| Periard et al. (97) | Qatar | Cross over | Healthy |
| Phillips et al. (98) | USA | Cross-sectional | Healthy |
| Place et al. (99) | France | Cross-sectional | Healthy |
| Saldanha et al. (100) | Sweden | Cross-sectional | Healthy |
| Sara et al. (a) (15) | USA | Cross-sectional | Healthy |
| Sara et al. (b) (33) | USA | Quasi-experimental | Achilles tendinopathy |
| Scaglioni et al. (a) (101) | Italy | Quasi-experimental | Healthy |
| Scaglioni et al. (b) (51) | France | Quasi-experimental | Healthy |
| Scaglioni et al. (c) (102) | France | Cross-sectional | Healthy |
| Shimoda et al. (103) | Japan | Randomised control trial | Healthy |
| Siegler et al. (104) | Australia | Quasi-experimental | Healthy |
| Simoneau et al. (105) | France | Cross-sectional | Healthy |
| Stutzig et al. (106) | Germany | Quasi-experimental | Healthy |
| Suzuki et al. (107) | Japan | Cross-sectional | Healthy |
| Trajano et al. (108) | Australia | Randomised control trial | Healthy |
| Unhjem et al. (109) | Norway | Cross-sectional | Healthy |
| Weir et al. (110) | Canada | Quasi-experimental | Healthy |

**Supplementary file E. Individual study participant position**

| **Study** | **Force transducer** | **Force sampling rate (Hz)** | **EMG** | **Knee joint position** | **Ankle joint position** | **Restraints** |
| --- | --- | --- | --- | --- | --- | --- |
| Akagi et al. (a) (52) | Isokinetic dynamometer | 2000 | MG, LG, SOL, TA | Full extension | Neutral | The participant’s pelvis and ankle were secured on the reclining seat and the foot pedal adapter of the dynamometer with non-elastic straps and/or a seat belt. |
| Akagi et al. (b) (38) | Isokinetic dynamometer | 4000 | NR | Full extension | Neutral | The participant’s torso, pelvic and ankle were secured on the reclining seat with elastic straps and seat belt |
| Avela et al. (53) | Ergometer chair | 1000-3000 | LG, SOL | 60 degrees flexion | Neutral | NR |
| Barber et al. (54) | Isokinetic dynamometer | 2000 | MG, LG, SOL, TA | Full extension | Neutral | Custom adjustable foot restraint system secured foot to minimise heel lift from footplate during contractions |
| Behm et al. (55) | Modified boot apparatus | 2000 | SOL, TA | 90 degrees flexion | Neutral | Legs secured in a modified boot apparatus |
| Cannavan et al. (56) | Isokinetic dynamometer | 2000 | MG, SOL, TA | Full extension | Neutral | Inelastic straps tightly placed across the chest, hips, thigh and foot |
| Cattagni et al. (a) (57) | Strain gauge | 2000 | SOL, MG, LG | Full extension | Neutral | Foot secured by straps |
| Cattagni et al. (b) (58) | Isokinetic dynamometer | 2000 | SOL, MG | 20 degrees flexion | Neutral | NR |
| Crivelli et al. (59) | Strain gauge | 2000 | TA | 90 degrees flexion | Neutral | Right foot secured with two Velcro bands to a custom footplate |
| Crouzier et la. (32) | Isokinetic dynamometer | 1000 | MG, LG, SOL | Full extension | Neutral | Foot was fixed with a rigid strap |
| Cruz et al. (60) | Load cell | 2000 | SOL | 90 degrees flexion | Neutral | A padded limb support above the knee and an ankle- level belt were used during contractions. |
| Dalton et al. (a) (61) | Isokinetic dynamometer | 2000 | SOL, MG, TA | 90 degrees flexion | 10 degrees dorsiflexion | Participant was fastened securely to the seat of the Biodex with inelastic straps around the shoulders and waist. A thigh support with an inelastic strap was used for stabilization of the limb and to minimize involvement of the thigh muscles. |
| Dalton et al. (b) (17) | Isokinetic dynamometer | 100 | NR | 90 degrees flexion and 10 degrees flexion | Neutral | Two Velcro straps across the toes and dorsum of the foot. Participants were fastened securely to the seat of the Biodex with inelastic straps around the shoulders and waist. A thigh support with an inelastic strap was used for stabilization of the limb and to minimize involvement of the thigh muscles. |
| Dalton et al. (c) (62) | Custom build isometric dynamometer | 500 | SOL, MG | 90 degrees flexion | Neutral | Leg was secured with a C-clamp pressing firmly against the distal aspect of the right thigh to minimise extraneous hip and knee movement |
| Dalton et al. (d) (16) | Isokinetic dynamometer | 100 | NR | 90 degrees flexion and 10 degrees flexion | Neutral | Foot was secured to the foot plate using a heel binding with an adjustable strap and participants were secured to the chair with straps around the shoulders and waist |
| Ekblom et al. (63) | Isokinetic dynamometer | 5000 | SOL | Full extension | Neutral | Shoulder pads and a broad Velcro strap over the right thigh of the participant were applied to prevent extraneous movements. |
| Ema et al. (18) | Isokinetic dynamometer | 500 | MG, LG, SOL, TA | Full extension and 90 degrees flexion | Neutral | Participants were secured at the body to the dynamometer with non- elastic straps |
| Fimland et al. (36) | Isometric dynamometer | 2000 | SOL, MG, LG, TA | Full extension | Neutral | Rigid straps made sure the heel and forefoot were secured to the footplate |
| Girard et al. (a) (64) | Dynamometric pedal | 2000 | SOL | 90 degrees flexion | Neutral | The foot of the leg performing the MVC was secured to the dynamometric pedal with three restraining straps |
| Girard et al. (b) (65) | Dynamometric pedal | 2000 | NR | 90 degrees flexion | Neutral | Foot securely strapped on the pedal by three straps |
| Gondin et al. (a) (66) | Strain gauge | 2000 | SOL, MG, LG | 60 degrees flexion | Neutral | Foot secured to pedal |
| Gondin et al. (b) (67) | Isokinetic dynamometer | 5000 | SOL, MG, LG | 90 degrees flexion | Neutral | To minimize hip and thigh motion during the contractions, and therefore to avoid the contribution of muscles other than the plantar flexors (e.g., knee extensors, hip flexors), straps were fastened across the chest and pelvis. The foot was also secured to the footplate by two straps. One strap was placed around the ankle and the second strap was placed around the foot, 1–2 cm proximal to the metatarsophalangeal joint of the toes. |
| Green et al. (68) | Custom build ergometer | NR | GM, TA | Full extension | Neutral |  |
| Grospretre et al. (69) | Constraint gauge | 2000 | TA, VL, SOL, GM, GL | 90 degrees flexion | Neutral | The ankle was firmly strapped to a pedal equipped with a constraint gauge |
| Guette et al. (70) | Strain gauge | 2000 | SOL, MG, LG, TA | 60 degrees flexion | Neutral | Dominant foot was fixed to pedal by two straps. |
| Hali et al. (19) | Isokinetic dynamometer | 500 | TA, MG, LG, SOL | 90 degrees flexion | 20 degrees dorsiflexion and 20 degrees plantar flexion | The foot was secured to the dynamometer using two inelastic straps across the toes and dorsum of the foot and one at the ankle. A metal C-shaped bar pressing firmly against the distal aspect of the thigh minimized extraneous leg and hip movement during the contractions. |
| Hartman et al. (71) | Isokinetic dynamometer | 2000 | MG, SOL | Full extension | Neutral | Restraining straps over the pelvis, trunk and thigh. Foot was secured in a heel cup attached to a footplate with toe and ankle straps over the metatarsals and malleoli |
| Harwood et al. (72) | Linear calibrated force transducer | 500 | SOL, LG | 90 degrees flexion | 10 degrees plantar flexion | the foot firmly secured with restraints to the foot plate of the dynamometer. |
| Herda et al. (73) | Load cell | 2000 | SOL, MG | Full extension | NR | Restraining straps over the pelvis and thigh |
| Hirata et al. (a) (74) | Isokinetic dynamometer | 2000 | NR | Full extension | Neutral | pelvis and ankle were secured on the dynamometer bed and the foot pedal adapter of the dynamometer with non-elastic straps and/or a seat belt, respectively |
| Hirata et al. (b) (75) | Isokinetic dynamometer | 2000 | SOL, MG, LG | NR | Neutral | The right foot was fixed to the dynamometer foot plate with nonelastic straps |
| Hoffman et al. (76) | Torque transducer | 2000 | SOL, MG | 5 degrees flexion | Neutral | The right foot strapped to a foot plate that was attached to a torque transducer. |
| Hotta et al. (77) | Load cell | 1000 | NR | Full extension | 10 degrees plantar flexion | A belt was used as a support to keep the hip joint, knee joint, and thigh unchanged during testing. The foot was also tightly secured by two straps to keep the ankle joint unchanged. |
| Jo et al. (78) | Isokinetic dynamometer | NR | NR | Full extension | Neutral | Feet, chest and thighs were secured with non-elastic straps |
| Kawakami et al. (79) | Electrical myometer | 1000 | SOL, MG, LG | Full extension and 90 degrees flexion | 10 degrees dorsiflexion | Waist, shoulders, trunk and thighs secured by Velcro bands |
| Kennedy et al. (80) | Isokinetic dynamometer | 4000 | SOL, MG, LG | Full extension | 10 degrees plantar flexion | The torso, waist and right thigh were strapped to the dynamometer chair to ensure that the participant’s body position did not change throughout the experiment. |
| Kirk et al. (81) | isometric dynamometer | 500 | SOL, MG, LG, TA | Full extension | Neutral | Two Velcro straps over the dorsum of the foot and c clamp pressed firmly against the distal aspect of the thigh |
| Klass et al. (82) | Strain gauge | 2500 | SOL, MG | Full extension | Neutral | The first strap was placed over the dorsum of the foot, and the second one was attached around the ankle and the calcaneum. |
| Klein et al. (35) | Custom built dynamometer | 500 | TA, LG, MG, SOL | 90 degrees flexion | 10 degrees dorsiflexion | The knee was stabilized from above with a U-shaped clamp. The foot was secured on the steel plate of the device with two Velcro straps: one across the instep, and the other 1 cm proximal to the great toe. |
| Knarr et al. (34) | Isokinetic dynamometer | NR | NR | Full extension | Neutral | Velcro straps were used to hold the foot and shank in position. Restraints were placed on the shoulders of the participant |
| Koryak et al. (a) (83) | Steal ring dynamometer | NR | NR | 90 degrees flexion | Neutral | Firmly secured |
| Koryak et al. (b) (37) | Steal ring dynamometer | NR | SOL | 90 degrees flexion | Neutral | the position of the seat was adjusted to the individual and then firmly secured. A rigid leg fixation ensured isometric conditions for the muscle contraction. |
| Kubo et al. (a) (84) | Isokinetic dynamometer | NR | MG, LG, SOL, TA | Full extension | Neutral | Waist and shoulders secured by adjustable lap belts |
| Kubo et al. (b) (85) | Electrical dynamometer | NR | NR | Full extension | Neutral | The waist and shoulders were secured by adjustable lap belts and were held in position |
| Kuchinad et al. (86) | Strain gauge | 2500 | SOL | 90 degrees flexion | Neutral | An upright adjustable chair was used to support the subject, with a belt secured across the hips to keep the pelvis from shifting while seated. The lower limb was locked in place with an adjustable steel frame to eliminate compensatory movement during the experiment |
| Lapole et al. (a) (87) | Portable ergometer | 2000 | NR | 20 degrees flexion | Neutral | Foot firmly strapped to footplate |
| Lapole et al. (b) (88) | Portable ergometer | NR | NR | 60 degrees flexion | Neutral |  |
| Maffiuletti et al. (89) | Strain gauge | 2000 | SOL, MG, LG, TA | 90 degrees flexion | Neutral | Foot secured to pedal |
| Marathamuthu et al. (90) | Isokinetic dynamometer | 2000 | MG | Full extension | Neutral | Ankle secured with straps |
| Merlet et al. (20) | Isokinetic dynamometer | NR | SOL, GM | Full extension, 90 degrees flexion and 30 degrees flexion | Neutral | The participants were securely stabilized by two crossover shoulder harnesses and a belt across the abdomen. |
| Miller et al. (91) | Isokinetic dynamometer | 2000 | SOL | Full extension | 15 degrees dorsiflexion and 20 degrees plantar flexion | Restraining straps over thigh, pelvis and trunk |
| Morse et al. (a) (92) | Isokinetic dynamometer | 1000 | TA | Full extension | 20 degrees dorsiflexion | Straps were used about the hip to prevent forward displacement of the body |
| Morse et al. (b) (21) | Isokinetic dynamometer | NR | MG, LG | Full extension | 15 degrees dorsiflexion, neutral and 20 degrees plantar flexion | Foot was secured tightly to footplate |
| Neyroud et al. (a) (93) | Strain gauge | 2000 | SOL, MG, LG, TA | Full extension | Neutral | Foot securely strapped at the level of the ankle and metatarsi to a pedal fixed to a wall. Straps were applied at the hip level. |
| Neyroud et al. (b) (94) | Strain gauge | 2000 | SOL, LG, MG | 90 degrees flexion | Neutral | To limit the contribution of muscle groups other than plantar flexors, the thigh was clamped down to the chair proximal to the knee, while harnesses limited upper body movements. |
| Nordlund et al. (95) | Force transducer | 5000 | SOL, MG, TA | Full extension | Neutral | The pelvis was strapped to the bench to keep the knee in a straight position and prevent adverse movements during the protocol. |
| Paris et al. (96) | Isokinetic dynamometer | 1000 | NR | Full extension | 10 degrees dorsiflexion | The right foot (self-reported dominant for all participants) was secured firmly to the footplate using an inelastic ratchet strap over the dorsum of the foot. Participants were further secured to the table using two large Velcro straps across the thighs and torso. |
| Periard et al. (97) | Dynamometric pedal | 2000 | NR | 100 degrees flexion | Neutral | Foot was secured with two restraining straps |
| Phillips et al. (98) | Isokinetic dynamometer | 1000 | SOL, MG, LG | 30 degrees flexion | Neutral | Restraining straps were placed over the chest, pelvis, and thigh, |
| Place et al. (99) | Isokinetic dynamometer | 2000 | SOL, MG, LG, TA | 60 degrees flexion | Neutral | straps were fastened across the chest and pelvis. The foot was also secured to the footplate by two straps. One strap was placed around the ankle and the second strap was placed around the foot, 1–2 cm proximal to the metatarsophalangeal joint of the toes |
| Saldanha et al. (100) | Force transducer | 1000 | SOL | Full extension | Neutral | Foot was tightly strapped to a footplate |
| Sara et al. (a) (15) | Isokinetic dynamometer | NR | SOL, MG, LG, TA | 90 degrees flexion | Neutral | Straps were placed across the waist, chest, and right thigh. Two straps were used around the ankle and one around the forefoot to maintain the plantar surface of the foot in contact with the foot plate |
| Sara et al. (b) (33) | Isokinetic dynamometer | NR | SOL, MG, LG, TA | Full extension | Neutral | Straps were placed across the waist, chest, and thigh. Two straps were secured around the ankle and one around the forefoot to maintain the plantar surface of the foot in contact with the foot plate |
| Scaglioni et al. (a) (101) | Isokinetic dynamometer | 1000 | SOL | Full extension | 20 degrees dorsiflexion | The pelvis was firmly fixed to the examination table to prevent movements during efforts. A strap was placed around the foot to secure it sturdily to the strain gauge of the dynamometer |
| Scaglioni et al. (b) (51) | Strain gauge | 2000 | SOL | 90 degrees flexion | Neutral | A strap was placed around the foot to secure it firmly to the pedal and thus create an effective mechanic constriction. |
| Scaglioni et al. (c) (102) | Custom made pedal with Strain gauge | 2000 | NR | 90 degrees flexion | Neutral | A strap was placed around the foot to secure it firmly to the pedal and thus create an effective mechanic constriction. |
| Shimoda et al. (103) | Isokinetic dynamometer | 1000 | MG, LG SOL | Full extension | Neutral | Straps around the hips and right thigh to minimize excessive body motion |
| Siegler et al. (104) | Custom build ergometer | 1000 | SOL, MG | Full extension | 5 degrees dorsiflexion |  |
| Simoneau et al. (105) | Isokinetic dynamometer | 2000 | SOL, MG, LG, TA | Full extension | Neutral | Waist was stabilised by means of a belt and foot was secured with a strap |
| Stutzig et al. (106) | Plantar flexor device | 2000 | SOL, LG, MG | Full extension and 80 degrees flexion | Neutral | NR |
| Suzuki et al. (107) | Isokinetic dynamometer | 10000 | VL, GM, BF, TA, FL, MG, LG, SOL | Full extension | Neutral | Straps placed around the shoulders, hips, right knee, and left ankle to minimise changes in joint angles |
| Trajano et al. (108) | Isokinetic dynamometer |  | SOL, LG | Full extension | Neutral | NR |
| Unhjem et al. (109) | Force platform | 2000 | SOL, GM, GL, TA | 90 degrees flexion | 20 degrees dorsiflexion | NR |
| Weir et al. (110) | Strain gauge | 6000 | SOL, MG, TA | 90 degrees flexion | Neutral | An adjustable knee clamp applied a downward pressure on the test limb, which eliminated lateral movements of the lower leg and prevented the heel from rising off the footplate during plantar flexion. |

NR, not reported; EMG, electromyography; MG, medial gastrocnemius; LG, lateral gastrocnemius; SOL, soleus; TA, tibialis anterior; VL, vastus lateralis; BF, biceps femoris; VM, vastus medialis.

**Supplementary file F. Individual study stimulation parameters**

| **Study** | **Stimulation location** | **Stimulus shape** | **Stimulus type** | **Stimulus duration (μs)** | **Interstimulus interval (ms)** | **Stimulation intensity (mA)** | **Stimulation frequency (Hz)** | **Stimulus voltage (V)** | **Supramaximal stimulation intensity (% above maximum)** | **VA Method (ITT, CAR)** |
| --- | --- | --- | --- | --- | --- | --- | --- | --- | --- | --- |
| Akagi et al. (a) (43) | Tibial nerve in popliteal fossa | Rectangular | Triplet | 200 | 10 | NR | 100 | NR | 20 | ITT |
| Akagi et al. (b) (26) | Tibial nerve in popliteal fossa | Rectangular | Single | 1000 | - | NR | NR | NR | 20 | ITT |
| Avela et al. (44) | Tibial nerve in popliteal fossa | Rectangular | Doublet | 1000 | NR | NR | 100 | NR | 25 | ITT |
| Barber et al. (45) | Tibial nerve in popliteal fossa | Square | Doublet | 100 | 10 | 20-150 | NR | 400 | 25 | ITT |
| Behm et al. (46) | Superior aspect of gastrocnemius | Rectangular | Doublet | 50-100 | 10 | 10-1000 | NR | 100 | NR | ITT |
| Cannavan et al. (47) | Proximal and distal ends of the triceps surae | NR | Doublet | 10 | NR | NR | 100 | NR | 40 | ITT |
| Cattagni et al. (a) (48) | Tibial nerve in popliteal fossa | Rectangular | Single | 1000 | - | NR | NR | 400 | 20 | ITT |
| Cattagni et al. (b) (49) | Posterior tibial nerve in the popliteal fossa | NR | Doublet | 1000 | NR | NR | 100 | 400 | 20 | ITT |
| Crivelli et al. (50) | Tibial nerve in popliteal fossa | Square | Single and Doublet | 1000 | 10 | 76(45) | NR | NR | 10 | ITT |
| Crouzier et la. (20) | Tibial nerve in popliteal fossa | NR | Doublet | 1000 | 10 | 103.4(36.9) | NR | 400 | 20 | ITT |
| Cruz et al. (51) | Tibial nerve in popliteal fossa | Square | Single | 200 | - | NR | NR | 400 | 30 | ITT |
| Dalton et al. (a) (52) | Tibial nerve in popliteal fossa | Square | Single | 100 | - | 378(92) | NR | 400 | 10-15 | ITT |
| Dalton et al. (b) (38) | Popliteal fossa | Square | Single | 100 | - | NR | NR | 400 | 10-15 | ITT |
| Dalton et al. (c) (53) | Tibial nerve in popliteal fossa | Square | Single | 100 | - | 250-600 | NR | 400 | 10-15 | ITT |
| Dalton et al. (d) (37) | Tibial nerve in popliteal fossa | Square | Single | 100 | - | 240-850 | NR | 400 | 20 | ITT |
| Ekblom et al. (54) | Tibial nerve in popliteal fossa | Square | Single | 1000 | - | NR | NR | NR | 50 | ITT |
| Ema et al. (39) | Tibial nerve in popliteal fossa | Rectangular | Single | 1000 | - | NR | NR | NR | 20 | ITT |
| Fimland et al. (24) | Tibial nerve in popliteal fossa | Square | NR | 1000 | NR | NR | NR | NR | 100mA | ITT |
| Girard et al. (a) (55) | Tibial nerve in popliteal fossa | Rectangular | Single | 200 | - | 85(22), 48-120 | NR | 400 | 50 | ITT |
| Girard et al. (b) (56) | Tibial nerve in popliteal fossa | Rectangular | Single | 200 | - | 130(31) | NR | 400 | 50 | ITT |
| Gondin et al. (a) (57) | Tibial nerve in popliteal fossa | Rectangular | Doublet | 1000 | 10 | NR | 20-100 | NR | NR | ITT, CAR |
| Gondin et al. (b) (58) | Posterior tibial nerve in popliteal fossa | Rectangular | Single | 1000 | - | 32-120 | 75 | NR | 50 | ITT |
| Green et al. (59) | Tibial nerve in popliteal fossa | Square | Single | 100 | - | 90-210 | NR | NR | 20 | ITT |
| Grospretre et al. (60) | Tibial nerve in popliteal fossa | Rectangular | Single | 1000 | - | NR | NR | NR | 20 | ITT |
| Guette et al. (61) | Posterior nerve in popliteal fossa | Rectangular | Doublet | 1000 | NR | 0-100 | NR | 400 | NR | ITT |
| Hali et al. (62) | Tibial nerve in popliteal fossa | Square | Single | 200 | - | 60-120 | NR | 400 | 20 | ITT |
| Hartman et al. (63) | Tibial nerve in popliteal fossa | Rectangular | Doublet | 1000 | NR | 30-300 | NR | NR | 20 | ITT, CAR |
| Harwood et al. (64) | Tibial nerve in popliteal fossa | Square | Doublet | 100 | NR | NR | NR | 200 | NR | ITT |
| Herda et al. (65) | Tibial nerve in popliteal fossa | Square | Doublet | 200 | NR | NR | 100 | 400 | 20 | ITT at 90% MVC |
| Hirata et al. (a) (66) | Tibial nerve in popliteal fossa | Rectangular | Triplet | 200 | 10 | NR | 100 | NR | 20 | ITT |
| Hirata et al. (b) (67) | Tibial nerve in popliteal fossa | Rectangular | NR | 200 | NR | NR | NR | NR | 20 | ITT |
| Hoffman et al. (68) | Popliteal fossa | NR | Doublet | NR | 15 | NR | NR | NR | 20 | ITT |
| Hotta et al. (69) | Tibial nerve in popliteal fossa | Square | Single | 1000 | - | NR | NR | NR | 20 | ITT |
| Jo et al. (70) | Tibial nerve in popliteal fossa | Rectangular | Single and Doublet | 200 | 10 | NR | NR | NR | 10-15 | ITT |
| Kawakami et al. (71) | Tibial nerve in popliteal fossa | Square | Doublet | 500 | 10 | NR | NR | NR | NR | ITT |
| Kennedy et al. (72) | Tibial nerve in popliteal fossa | NR | Single | 200 | - | 173(30.5) | NR | NR | NR | ITT |
| Kirk et al. (73) | Tibial nerve in popliteal fossa | Square | Single | 100 | - | NR | NR | 400 | 15 | ITT |
| Klass et al. (74) | Tibial nerve in popliteal fossa | Rectangular | Single and Doublet | 1000 | 10 | NR | NR | NR | 10-20 | ITT |
| Klein et al. (22) | Tibial nerve in popliteal fossa | NR | Single | 50-100 | - | NR | 25-100 | NR | 20 | ITT |
| Knarr et al. (23) | NR | Square | Single | 600 | - | NR | 100 | 135 | NR | CAR |
| Koryak et al. (a) (75) | Tibial nerve in popliteal fossa | Rectangular | NR | 1000 | NR | NR | 150 | NR | 30-40 | NR |
| Koryak et al. (b) (25) | Tibial nerve in popliteal fossa | Rectangular | NR | 1000 | NR | NR | NR | NR | NR | NR |
| Kubo et al. (a) (76) | Tibial nerve in popliteal fossa | Rectangular | Triplet | 500 | 10 | NR | NR | NR | NR | ITT |
| Kubo et al. (b) (77) | Tibial nerve in popliteal fossa | Rectangular | Triplet | 500 | 10 | NR | NR | NR | NR | ITT |
| Kuchinad et al. (78) | Tibial nerve in popliteal fossa | Square | Doublet | 50-100 | NR | NR | 100 | NR | 20 | ITT |
| Lapole et al. (a) (79) | Tibial nerve in popliteal fossa | Rectangular | Single | 1000 | - | NR | NR | NR | 25 | ITT |
| Lapole et al. (b) (80) | Tibial nerve in popliteal fossa | NR | Single | 1000 | - | NR | NR | NR | 20 | ITT |
| Maffiuletti et al. (81) | Tibial nerve in popliteal fossa | Rectangular | Single | 1000 | - | NR | NR | NR | 10 | ITT |
| Marathamuthu et al. (82) | Tibial nerve in popliteal fossa | NR | NR | 200 | NR | 45-120 | NR | NR | 20 | ITT, CAR |
| Merlet et al. (40) | Tibial nerve in popliteal fossa | Rectangular | Single | 1000 | - | NR | NR | 400 | 10 | ITT |
| Miller et al. (83) | Tibial nerve | Square | Doublet | 1000 | NR | NR | 100 | NR | 20 | ITT |
| Morse et al. (a) (84) | Distal to the popliteal crease | Square | Doublet | 50 | 10 | NR | NR | NR | NR | ITT |
| Morse et al. (b) (85) | Tibial nerve in popliteal fossa | NR | Doublets | 50 | 10 | 50-100 | NR | NR | NR | ITT |
| Neyroud et al. (a) (86) | Tibial nerve in popliteal fossa | Rectangular | Single and Doublet | 1000 | NR | 157(37), 120-228 | NR | 400 | 20 | ITT |
| Neyroud et al. (b) (87) | tibial nerve | Rectangular | Doublet | 1000 | NR | NR | 100 | 400 | 20 | ITT |
| Nordlund et al. (88) | Tibial nerve in popliteal fossa | NR | Single | 1000 | - | NR | NR |  | 50 | ITT |
| Paris et al. (89) | Tibial nerve in popliteal fossa | Square | Single | 100 | - | 89.5(17.8) | NR | 400 | 20 | ITT |
| Periard et al. (90) | Tibial nerve in popliteal fossa | Square | Doublet | 200 | NR | NR | NR | 400 | 50 | ITT |
| Phillips et al. (91) | Tibial nerve in popliteal fossa | Square | Singlet | 100 | - | NR | NR | NR | 20 | ITT |
| Place et al. (92) | Tibial nerve in popliteal fossa | Rectangular | Doublet | 1000 | 10 | 40-100 | NR | NR | 50 | ITT |
| Saldanha et al. (93) | Tibial nerve in popliteal fossa | Rectangular | Doublet | 500 | 20 | NR | NR | NR | NR | ITT |
| Sara et al. (a) (13) | Tibial nerve in popliteal fossa | Square | Doublet | 100 | NA | NR | 100 | 400 | 10 | ITT |
| Sara et al. (b) (21) | Tibial nerve in popliteal fossa | Square | Single | 100 | - | NR | NR | 400 | 10 | ITT |
| Scaglioni et al. (a) (94) | Belly of gastrocnemius muscle | Square | Single | 50 | - | NR | 1 | NR | NR | NR |
| Scaglioni et al. (b) (42) | Tibial nerve in popliteal fossa | Square | Single | 1000 | - | 50-86 | NR | 400 | NR | ITT |
| Scaglioni et al. (c) (95) | Tibial nerve in popliteal fossa | Square | Single | 1000 | - | 50-86 | NR | 400 | 10 | ITT |
| Shimoda et al. (96) | Tibial nerve in popliteal fossa | NR | Triplet | NR | 10 | NR | NR | NR | 10-20 | ITT |
| Siegler et al. (97) | Posterior tibial nerve in popliteal fossa | Square | Single | 1000 | - | 70-95 | NR | 400 | 50 | ITT |
| Simoneau et al. (98) | Tibial nerve in popliteal fossa | Rectangular | Doublet | 1000 | NR | NR | 100 | 400 | 0 | ITT |
| Stutzig et al. (99) | Tibial nerve in popliteal fossa | Rectangular | Doublet | 1000 | 10 | NR | NR | NR | 20 | ITT |
| Suzuki et al. (100) | Tibial nerve at the level of the popliteal fossa | NR | Doublet | 200 | NR | 101.3(47.8) | NR | NR | 10 mA | ITT |
| Trajano et al. (101) | Tibial nerve in popliteal fossa | Square | Single | 1000 | - | NR | NR | NR | 20 | ITT |
| Unhjem et al. (102) | Tibial nerve in popliteal fossa | Square | Single | 1000 | - | NR | NR | NR | 50 | ITT |
| Weir et al. (103) | Posterior tibial nerve | Square | Doublet | NR | 10 | NR | NR | NR | 50 | ITT |

All data reported as mean (SD); NR, not reported; ITT, interpolated twitch technique; CAR, central activation ratio.

**Supplementary file G. Individual study demographic information**

| **Study** | **Group** | **Sample size** | **Sex (female)** | **Age** | **Height** | **Weight** |
| --- | --- | --- | --- | --- | --- | --- |
| Akagi et al. (a) | Healthy | 12 | 0 | 22 (1) | 170 (3.9) | 64.7 (13.3) |
| Akagi et al. (b) | Young men | 20 | 0 | 22 (2) | 170.6 (5) | 62.6 (6.5) |
|  | Young women | 20 | 20 | 22 (1) | 157.4 (4.1) | 51.7 (6.5) |
|  | Older men | 19 | 0 | 73 (5) | 165.4 (6.5) | 67.6 (10.3) |
|  | Older women | 14 | 14 | 72 (7) | 154.5 (4.7) | 56 (5.9) |
| Avela et al. | Healthy | 8 | 0 | 25 | NR | NR |
| Barber et al. | Older adults | 16 | 7 | 70 (3) | 166 (8) | 73 (18) |
|  | Younger adults | 18 | 8 | 27 (3) | 178 (9) | 71 (13) |
| Behm et al. | Resistance trained | 14 | 7 | 21.5 (4) | 169.3 (6.3) | 65.4 (5.2) |
|  | Untrained controls | 14 | 7 | 24.4 (5) | 167.9 (6.3) | 68.1 (7.1) |
| Cannavan et al. | Healthy | 9 | 0 | 20.4 (0.7) | 179.4 (6.3) | 76 (8) |
| Cattagni et al. (a) | Non-fallers | 23 | 17 | 83.3 (3.9) | 159.9 (7.6) | 61.8 (9.9) |
|  | Fallers | 25 | 18 | 84 (4.1) | 161.2 (9.8) | 63.4 (9.5) |
| Cattagni et al. (b) | Healthy | 10 | 0 | 22.6 (4) | 171.5 (11.9) | 67.5 (13.4) |
| Crivelli et al. | Healthy | 10 | 0 | 31 (5) | 180 (5) | 76 (6) |
| Crouzier et al. | Achilles tendinopathy | 21 | 3 | 36.2 (8.3) | 175.9 (8) | 72.7 (8.7) |
|  | Healthy | 21 | 3 | 35.1 (7.7) | 177.5 (7.8) | 71.8 (10.5) |
| Cruz et al. | Healthy | 10 | 0 | 27 (5) | 176 (7) | 79 (14) |
| Dalton et al. (a) | Older men | 10 | 0 | 77.5 (3) | 175.5 (7.9) | 88.6 (13.1) |
|  | Young men | 10 | 0 | 24.1 (2.8) | 175 (8.7) | 78.6 (7.9) |
| Dalton et al. (b) | Knee flexion | 11 | 0 | 26.3 (3.4) | 178.1 (6.1) | 80.1 (3.4) |
|  | Knee extension | 11 | 0 | 26.3 (3.4) | 178.1 (6.1) | 80.1 (3.4) |
| Dalton et al. (c) | Older men | 6 | 0 | 75.3 (4.1) | 177 (4.2) | 88 (12.2) |
|  | Young men | 6 | 0 | 23.5 (2.9) | 173.7 (8.5) | 82.3 (11.8) |
| Dalton et al. (d) | Young men | 10 | 0 | 26.3 (3.4) | 178.1 (6.1) | 80.1 (3.4) |
|  | Older men | 10 | 0 | 77.6 (4.4) | 175.7 (3.8) | 83.7 (11.3) |
| Ekblom et al. | Training group | 9 | 4 | 27 (4.8) | 180 (9) | 73.8 (14.8) |
|  | Control group | 11 | 4 | 27 (6) | 177 (10) | 70.6 (10) |
| Ema et al. | Older men | 17 | 0 | 74 (5) | 164.3 (6.2) | 67.6 (10.3) |
|  | Older women | 15 | 15 | 72 (4) | 154.4 (4.6) | 55.5 (6) |
| . | Young men | 18 | 0 | 22 (2) | 170.6 (5) | 62.6 (6.5) |
|  | Young women | 19 | 19 | 22 (1) | 157.1 (4) | 50.9 (5.6) |
| Fimland et al. | Stroke | 12 | 4 | 44.8 (36.5) | NR | NR |
| Girard et al.(a) | Healthy | 17 | 0 | 27 (1) | 184 (1) | 80.1 (1.6) |
| Girard et al. (b) | Healthy | 14 | 0 | 32 (4) | 178 (5) | 78 (8) |
| Gondin et al (a) | Intervention | 8 | 0 | 25.8 (1.6) | 176.4 (2) | 70 (2.6) |
|  | Control | 9 | 0 | 24.7 (1.1) | 181.3 (2.1) | 76.8 (3.1) |
| Gondin et al. (b) | Intervention | 12 | 0 | 21.7 (3.4) | 176.6 (7.4) | 70.5 (4.7) |
|  | Control | 7 | 0 | 26.6 (5.5) | 178.3 (8.4) | 71.9 (6.6) |
| Green et al. | Healthy | 6 | 2 | 32 | NR | NR |
| Grospretre et al. | Healthy | 12 | 1 | 25.4 (8) | 176 (6) | 62.1 (7.5) |
|  | Healthy | 12 | 6 | 29.6 (10.5) | 171 (7) | 64.2 (9.2) |
| Guette et al. | Healthy | 11 | 0 | 23.4 (0.7) | 177.7 (2.1) | 70 (2.2) |
| Hali et al. | Dorsiflexion | 10 | 0 | 24 (3) | 181 (5) | 81 (7) |
|  | Plantar flexion | 10 | 0 | 24 (3) | 181 (5) | 81 (7) |
| Hartman et al. | Untrained | 6 | 0 | 25.8 (4.9) | 177.1 (8.7) | 73.6 (8.7) |
|  | Trained | 8 | 0 | 25.7 (4.2) | 175.9 (7.7) | 78.1 (6.2) |
| Harwood et al. | Woman | 8 | 8 | 24 (2) | 164 (3) | 58 (4) |
|  | Men | 8 | 0 | 24 (2) | 187 (5) | 96 (18) |
| Herda et al. | Woman | 11 | 11 | 21 (2.5) | NR | 52 (6) |
|  | Men | 14 | 0 | 21 (2.5) | NR | 69 (9) |
| Hirata et al. (a) | Intervention 1 | 12 | 0 | 22 (1) | 176 (8) | 67 (6) |
|  | Intervention 2 | 12 | 0 | 23 (1) | 171 (6) | 65 (9) |
|  | Control | 12 | 0 | 22 (1) | 170 (4) | 65 (13) |
| Hirata et al. (b) | Young adults | 29 | 14 | 23 (3) | 165.3 (8.4) | 59.8 (11.9) |
|  | Older adults | 30 | 15 | 72 (5) | 159.8 (9) | 60.1 (11.9) |
| Hoffman et al. | Healthy | 10 | 3 | 29.5 (4.6) | 173.3 (7) | 72.6 (10.6) |
| Hotta et al. | Healthy | 11 | 0 | 21.6 (3.4) | 170.2 (5.7) | 60.8 (9.4) |
| Jo et al. | Men | 10 | 0 | 27.3 (5.3) | 183.5 (5.8) | 91.5 (11.4) |
|  | Woman | 14 | 14 | 29.4 (4.9) | 167.4 (7.3) | 63.9 (9.8) |
| Kawakami et al. | Healthy | 9 | 0 | 33.5 | 172 (4.2) | 71.1 (9.7) |
| Kennedy et al. | Men | 8 | 0 | 25 | 176.8 (4.9) | 71.8 (6.7) |
| Kennedy et al. | Woman | 6 | 6 | 25 | 170.5 (3.9) | 60.5 (4.5) |
| Kirk et al. | Young men | 10 | 0 | 27 (3) | 180 (6) | 81 (10) |
|  | Older men | 10 | 0 | 81 (4) | 171 (7) | 75 (14) |
| Klass et al. | Healthy | 10 | 4 | 25 | NR | NR |
| Klein et al. | Stroke | 7 | 2 | 55.8 (3.6) | NR | NR |
| Knarr et al. | Stroke | 17 | 2 | 60.7 (8.9) | NR | NR |
| Koryak et al. (a) | Healthy | 6 | 0 | 38 (4.9) | 179 (2.9) | 79.5 (6.9) |
| Koryak et al. (b) | Healthy | 4 | 4 | 31.5 (3.4) | 162.3 (3.8) | 55 (3.6) |
| Kubo et al. (a) | Intervention 1 | 10 | 0 | 22.3 (1.1) | 171.4 (6.1) | 63.8 (8.7) |
|  | Intervention 2 | 10 | 0 | 22.5 (1.6) | 169.8 (3.3) | 62.7 (7.8) |
| Kubo et al. (b) | 20-year olds | 19 | 0 | 24.5 (2.1) | 171.6 (3.9) | 69.7 (10.3) |
|  | 30-year olds | 15 | 0 | 35.2 (2.2) | 171.5 (4.8) | 72.4 (8.6) |
|  | 50-year olds | 10 | 0 | 51.4 (4.4) | 172.4 (4.3) | 73.9 (8) |
|  | 70-year olds | 15 | 0 | 69.7 (4.5) | 159 (6.3) | 59.4 (8) |
| Kuchinad et al. | Healthy | 11 | 1 | 36 | NR | NR |
| Lapole et al. (a) | Healthy | 10 | 0 | 23.3 (5) | 180 (7.5) | 75.2 (9.6) |
| Lapole et al. (b) | Healthy | 29 | 0 | 21.7 (1.7) | 177 (9) | 71.7 (14.1) |
| Maffiuletti et al. | Intervention | 8 | 0 | 20.4 (2.1) | 186.4 (8) | 83.5 (9.6) |
|  | Control | 6 | 0 | 26 (5.1) | 176.7 (4.8) | 69.5 (4.6) |
| Marathamuthu et al. | Healthy | 10 | 0 | 28.7 (4.24) | 170 (4) | 72.3 (6.53) |
| Merlet et al. | Healthy | 12 | 5 | 22.5 (1.2) | 172.5 (9.7) | 63.5 (9.2) |
| Miller et al. | Healthy | 14 | 0 | 22 (3) | 177 (6) | 84 (11) |
| Morse et al. (a) | Elderly | 19 | 0 | 73.83 (3.5) | 173.4 (4.4) | 78.4 (8.3) |
|  | Young | 12 | 0 | 25.3 (4.4) | 176.4 (7.7) | 79.1 (11.9) |
| Morse et al. (b) | Young men | 9 | 0 | 24.7 (4.7) | 179.3 (7.8) | 76.9 (12.4) |
|  | Elderly men | 9 | 0 | 83.7 (3.6) | 170.4 (4.5) | 77.5 (8.1) |
| Neyroud et al. (a) | Healthy | 10 | 3 | 35 (10) | 173 (6) | 71 (10) |
| Neyroud et al. (b) | Healthy | 14 | 3 | 27 (4) | 175 (5) | 72 (9) |
| Nordlund et al. | Healthy | 10 | 0 | 28 (5) | 179 (5) | 79 (5) |
| Paris et al. | Healthy | 13 | 2 | 24.2 (2.6) | NR | NR |
| Periard et al. | Healthy | 12 | 0 | 22 (4) | 183.5 (7.7) | 80.8 (9.5) |
| Phillips et al. | Middle aged adults | 22 | 22 | 54 (5.8) | 165.6 (5.9) | 72.2 (13) |
|  | Young adults | 17 | 17 | 21.8 (1.4) | 166.8 (7.1) | 70.4 (10.7) |
| Place et al. | Healthy | 12 | 0 | 28 (4.8) | 174.6 (6.3) | 68.8 (7.5) |
| Saldanha et al. | Healthy | 8 | 0 | 27.6 (2.4) | 182 (8.7) | 74.5 (0.7) |
| Sara et al. (a) | Men | 14 | 0 | 21.5 (1.8) | 181 (8) | 79.4 (10.3) |
|  | Woman | 14 | 14 | 21.1 (2.9) | 166 (7) | 64 (10.8) |
| Sara et al. (b) | Control | 14 | 7 | 23.6 (5.5) | 148 (11) | 67.4 (13) |
|  | Achilles tendinopathy | 14 | 4 | 31 (11.1) | 152 (9) | 87.7 (21.3) |
| Scaglioni et al. (a) | Healthy | 14 | 0 | 68 | 172 | 80 |
| Scaglioni et al. (b) | Healthy | 11 | 0 | 26 (4) | 178 (5) | 72 (7) |
| Scaglioni et al. (c) | Young men | 11 | 0 | 26 (4) | 178 (5) | 72 (7) |
|  | Older men | 13 | 0 | 76 (3) | 168 (4) | 73 (10) |
| Shimoda et al. | Intervention | 10 | 0 | 22 (1.1) | 170.9 (7.6) | 64.8 (7.4) |
|  | Control | 10 | 0 | 21.9 (0.7) | 174.2 (4.6) | 65.4 (7.2) |
| Siegler et al. | Healthy | 8 | 0 | 24 (2) | 179.3 (6.2) | 81.1 (11.9) |
| Simoneau et al. | Young adults | 11 | 0 | 23.9 (1.7) | 176 (4) | 74 (8.53) |
|  | Older adults | 12 | 0 | 77.1 (1.8) | 168 (3) | 74.5 (10.9) |
| Stutzig et al. | Healthy | 13 | 8 | 27.4 (5.1) | 174.1 (8.2) | 69.5 (12.6) |
| Suzuki et al. | Healthy | 8 | 0 | 22 (3) | 171.2 (6) | 68.1 (10.3) |
| Trajano et al. | Healthy | 13 | 0 | 26.5 (5) | 172 (9) | 71.1 (13.8) |
| Unhjem et al. | Sedentary older adults | 10 | 0 | 71 (4) | 179 (6) | 94.7 (12.9) |
|  | Recreationally active older adults | 11 | 0 | 73 (6) | 178 (8) | 87 (17.6) |
|  | Master older athletes | 11 | 0 | 71 (4) | 178 (9) | 92.5 (12.9) |
|  | Young adults | 9 | 0 | 22 (2) | 187 (5) | 79.3 (12) |
| Weir et al. | Healthy | 15 | 15 | 23.1 (2.1) | 165.8 (5.8) | 58.9 (3.8) |

NR, Not reported; all data displayed as mean (SD)

**Supplementary file H. Summary statistics for knee flexion**

|  | | **Healthy** | | | | | **Achilles tendinopathy** | | | | | **Stroke** | | | | |
| --- | --- | --- | --- | --- | --- | --- | --- | --- | --- | --- | --- | --- | --- | --- | --- | --- |
|  |  | Valid N | Mean | Median | Minimum | Maximum | Valid N | Mean | Median | Minimum | Maximum | Valid N | Mean | Median | Minimum | Maximum |
| **0˚ knee flexion** | Sample size | 65 | 13 | 12 | 6 | 25 | 2 | 18 | 18 | 14 | 21 | 2 | 15 | 15 | 12 | 17 |
|  | ITT (%) | 63 | 89.11 | 90.70 | 52.00 | 100.00 | 2 | 89.55 | 89.55 | 81.20 | 97.90 | 1 | 29.00 | 29.00 | 29.00 | 29.00 |
|  | CAR | 10 | 78.84 | 99.05 | .67 | 100.00 | 0 |  |  |  |  | 1 | .39 | .39 | .39 | .39 |
|  | MVC (Nm) | 54 | 116.21 | 120.00 | 34.70 | 229.00 | 2 | 128.6 | 128.60 | 121.10 | 136.10 | 2 | 51.95 | 51.95 | 33.90 | 70.00 |
| **5˚ knee flexion** | Sample size | 1 | 10 | 10 | 10 | 10 | 0 |  |  |  |  | 0 |  |  |  |  |
|  | ITT (%) | 1 | 97.12 | 97.12 | 97.12 | 97.12 | 0 |  |  |  |  | 0 |  |  |  |  |
|  | CAR | 0 |  |  |  |  | 0 |  |  |  |  | 0 |  |  |  |  |
|  | MVC (Nm) | 0 |  |  |  |  | 0 |  |  |  |  | 0 |  |  |  |  |
| **10˚ knee flexion** | Sample size | 3 | 10 | 10 | 10 | 11 | 0 |  |  |  |  | 0 |  |  |  |  |
|  | ITT (%) | 3 | 96.10 | 96.30 | 95.00 | 97.00 | 0 |  |  |  |  | 0 |  |  |  |  |
|  | CAR | 0 |  |  |  |  | 0 |  |  |  |  | 0 |  |  |  |  |
|  | MVC (Nm) | 3 | 183.33 | 192.30 | 154.20 | 203.50 | 0 |  |  |  |  | 0 |  |  |  |  |
| **20˚ knee flexion** | Sample size | 2 | 10 | 10 | 10 | 10 | 0 |  |  |  |  | 0 |  |  |  |  |
|  | ITT (%) | 2 | 94.38 | 94.38 | 94.10 | 94.65 | 0 |  |  |  |  | 0 |  |  |  |  |
|  | CAR | 0 |  |  |  |  | 0 |  |  |  |  | 0 |  |  |  |  |
|  | MVC | 2 | 146.63 | 146.63 | 125.85 | 167.40 | 0 |  |  |  |  | 0 |  |  |  |  |
| **30˚ knee flexion** | Sample size | 3 | 17 | 17 | 12 | 22 | 0 |  |  |  |  | 0 |  |  |  |  |
|  | ITT (%) | 3 | 91.57 | 92.70 | 88.80 | 93.20 | 0 |  |  |  |  | 0 |  |  |  |  |
|  | CAR | 0 |  |  |  |  | 0 |  |  |  |  | 0 |  |  |  |  |
|  | MVC (Nm) | 3 | 128.80 | 136.40 | 109.00 | 141.00 | 0 |  |  |  |  | 0 |  |  |  |  |
| **60˚ knee flexion** | Sample size | 6 | 13 | 10 | 8 | 29 | 0 |  |  |  |  | 0 |  |  |  |  |
|  | ITT (%) | 6 | 88.95 | 94.85 | 52.80 | 99.10 | 0 |  |  |  |  | 0 |  |  |  |  |
|  | CAR | 2 | 98.25 | 98.25 | 98.10 | 98.40 | 0 |  |  |  |  | 0 |  |  |  |  |
|  | MVC (Nm) | 6 | 144.43 | 135.90 | 117.00 | 214.80 | 0 |  |  |  |  | 0 |  |  |  |  |
| **80˚ knee flexion** | Sample size | 1 | 13 | 13 | 13 | 13 | 0 |  |  |  |  | 0 |  |  |  |  |
|  | ITT (%) | 1 | 89.80 | 89.80 | 89.80 | 89.80 | 0 |  |  |  |  | 0 |  |  |  |  |
|  | CAR | 0 |  |  |  |  | 0 |  |  |  |  | 0 |  |  |  |  |
|  | MVC (Nm) | 1 | 154.10 | 154.10 | 154.10 | 154.10 | 0 |  |  |  |  | 0 |  |  |  |  |
| **90˚ knee flexion** | Sample size | 47 | 11 | 11 | 4 | 19 | 0 |  |  |  |  | 1 | 7 | 7 | 7 | 7 |
|  | ITT (%) | 47 | 92.49 | 95.60 | 62.40 | 99.50 | 0 |  |  |  |  | 1 | 48.50 | 48.50 | 48.50 | 48.50 |
|  | CAR | 0 |  |  |  |  | 0 |  |  |  |  | 0 |  |  |  |  |
|  | MVC (Nm) | 39 | 143.99 | 141.00 | 85.20 | 284.80 | 0 |  |  |  |  | 1 | 56.70 | 56.70 | 56.70 | 56.70 |
| **100˚ knee flexion** | Sample size | 1 | 12 | 12 | 12 | 12 | 0 |  |  |  |  | 0 |  |  |  |  |
|  | ITT (%) | 1 | 95.90 | 95.90 | 95.90 | 95.90 | 0 |  |  |  |  | 0 |  |  |  |  |
|  | CAR | 0 |  |  |  |  | 0 |  |  |  |  | 0 |  |  |  |  |
|  | MVC (Nm) | 1 | 82.70 | 82.70 | 82.70 | 82.70 | 0 |  |  |  |  | 0 |  |  |  |  |

ITT, interpolated twitch technique; CAR, central activation ratio; MVC, maximal voluntary contraction; Nm, newton meters

**Supplementary file I. Methodological quality of included studies**

| **Author** | **Were the criteria for inclusion in the sample clearly defined?** | **Were the study participants and the setting described in detail?** | **Was the exposure measured in a valid and reliable way?** | **Were objective, standard criteria used for measurement of the condition?** | **Were confounding factors identified?** | **Were strategies to deal with confounding factors stated?** | **Were the outcomes measured in a valid and reliable way?** | **Was appropriate statistical analysis used?** | **Quality** |
| --- | --- | --- | --- | --- | --- | --- | --- | --- | --- |
| *Akagi et al. (a)* | n | y | u |  | n | n | y | y | Low |
| *Akagi et al. (b)* | n | y | y |  | n | y | y | y | Low |
| *Avela et al.* | n | n | y |  | n | n | n | y | Low |
| *Barber et al.* | n | y | y |  | n | y | y | y | Low |
| *Behm et al.* | n | y | n |  | n | n | n | y | Low |
| *Cannavan et al.* | n | y | y |  | n | n | y | y | Low |
| *Cattagni et al. (a)* | y | y | y |  | y | n | y | y | Low |
| *Cattagni et al. (b)* | n | y | y |  | n | n | y | n | Low |
| *Crivelli et al.* | n | y | u |  | n | n | y | y | Low |
| *Crouzier et al.* | y | y | y | y | n | n | y | y | Low |
| *Cruz et al.* | y | y | n |  | y | n | y | n | Low |
| *Dalton et al. (a)* | y | y | u |  | y | n | y | y | Low |
| *Dalton et al. (b)* | n | y | u |  | n | n | n | y | Low |
| *Dalton et al. (c)* | y | y | u |  | n | n | n | y | Low |
| *Dalton et al. (d)* | y | y | y |  | y | n | n | y | Low |
| *Ekblom et al.* | n | y | y |  | n | n | y | y | Low |
| *Ema et al.* | n | y | y |  | n | n | n | y | Low |
| *Fimland et al.* | y | n | y | y | y | n | y | y | Low |
| *Girard et al.* (a) | y | y | y |  | y | y | y | y | High |
| *Girard et al.* (b) | u | y | y |  | n | n | y | y | Low |
| *Gondin et al. (a)* | y | y | n |  | y | n | y | y | Low |
| *Gondin et al. (b)* | n | y | y |  | n | n | y | y | Low |
| *Green et al.* | y | n | y |  | n | n | n | n | Low |
| *Grospretre et al.* | y | y | y |  | y | n | y | y | Low |
| *Guette et al.* | n | y | n |  | n | n | y | y | Low |
| *Hali et al.* | y | y | y |  | y | n | y | n | Low |
| *Hartman et al.* | y | y | y |  | y | n | y | y | Low |
| *Harwood et al.* | y | y | n |  | y | n | y | y | Low |
| *Herda et al.* | y | n | y |  | y | n | n | y | Low |
| *Hirata et al. (a)* | y | y | y |  | y | n | y | y | Low |
| *Hirata et al. (b)* | y | y | y |  | y | y | y | y | High |
| *Hoffman et al.* | y | y | y |  | n | n | y | y | Low |
| *Hotta et al.* | n | y | y |  | n | n | n | y | Low |
| *Jo et al.* | y | y | u |  | y | y | y | y | Low |
| *Kawakami et al.* | n | y | n |  | n | n | u | y | Low |
| *Kennedy et al.* | n | y | n |  | n | n | y | y | Low |
| *Kirk et al.* | y | y | u |  | n | n | y | y | Low |
| *Klass et al.* | n | n | y |  | n | n | y | n | Low |
| *Klein et al.* | y | y | y | y | y | n | y | y | Low |
| *Knarr et al.* | n | n | n | n | n | n | n | y | Low |
| *Koryak et al. (a)* | n | y | n |  | n | n | n | n | Low |
| *Koryak et al. (b)* | n | y | n |  | n | n | n | n | Low |
| *Kubo et al. (a)* | n | y | n |  | n | n | n | y | Low |
| *Kubo et al. (b)* | n | y | n |  | n | n | n | y | Low |
| *Kuchinad et al.* | y | n | y |  | y | n | y | y | Low |
| *Lapole et al. (a)* | u | y | y |  | n | n | y | y | Low |
| *Lapole et al. (b)* | n | y | y |  | n | n | n | y | Low |
| *Maffiuletti et al.* | u | y | u |  | y | n | y | y | Low |
| *Marathamuthu et al.* | y | y | y |  | y | n | y | y | Low |
| *Merlet et al.* | y | y | y |  | y | n | y | y | Low |
| *Miller et al.* | y | y | y |  | y | n | y | y | Low |
| *Morse et al. (a)* | y | y | u |  | y | n | y | n | Low |
| *Morse et al. (b)* | y | y | n |  | y | n | n | y | Low |
| *Neyroud et al. (a)* | n | y | y |  | n | n | y | y | Low |
| *Neyroud et al. (b)* | n | y | y |  | n | n | y | y | Low |
| *Nordlund et al.* | n | y | y |  | n | n | y | y | Low |
| *Paris et al.* | n | n | y |  | n | n | n | y | Low |
| *Periard et al.* | n | y | y |  | n | n | y | y | Low |
| *Phillips et al.* | y | y | y |  | y | n | y | y | Low |
| *Place et al.* | u | y | y |  | n | n | y | y | Low |
| *Saldanha et al.* | n | y | n |  | n | n | n | y | Low |
| *Sara et al. (a)* | y | y | u |  | y | y | n | y | Low |
| *Sara et al. (b)* | y | y | u | y | y | y | u | y | Low |
| *Scaglioni et al. (a)* | y | y | n |  | n | n | n | y | Low |
| *Scaglioni et al. (b)* | y | y | n |  | y | n | y | y | Low |
| *Scaglioni et al. (c)* | y | y | u |  | y | n | y | y | Low |
| *Shimoda et al.* | y | y | u |  | y | n | n | y | Low |
| *Siegler et al.* | n | y | y |  | n | n | n | y | Low |
| *Simoneau et al.* | y | y | n |  | y | n | y | y | Low |
| *Stutzig et al.* | y | y | y |  | y | n | y | y | Low |
| *Suzuki et al.* | y | y | u |  | n | n | y | y | Low |
| *Trajano et al.* | y | y | y |  | y | n | n | y | Low |
| *Unhjem et al.* | y | y | y |  | n | n | y | y | Low |
| *Weir et al.* | y | y | n |  | n | n | y | y | Low |

**Supplementary file J. Egger's Regression-Based Test^a,b^**

|  | **Parameter** | **Coefficient** | **Std. Error** | **t** | **Sig. (2-tailed)** | **95% Confidence Interval** | |
| --- | --- | --- | --- | --- | --- | --- | --- |
|  |  |  |  |  |  | **Lower** | **Upper** |
| **Healthy** | Intercept | 101.208 | 1.4917 | 67.847 | <.001 | 98.254 | 104.161 |
|  | Standard error^c^ | -3.870 | 0.4283 | -9.034 | <.001 | -4.718 | -3.022 |
|  | Age | -0.062 | 0.0261 | -2.386 | 0.019 | -0.114 | -0.011 |
|  | Knee position | 0.004 | 0.0126 | 0.329 | 0.743 | -0.021 | 0.029 |
|  | Ankle position | -0.072 | 0.0762 | -0.949 | 0.345 | -0.223 | 0.079 |
| **Overall** | Intercept | 101.817 | 1.4665 | 69.428 | <0.001 | 98.914 | 104.719 |
|  | Standard error^c^ | -4.180 | 0.3919 | -10.668 | <.001 | -4.956 | -3.405 |
|  | Age | -0.064 | 0.0267 | -2.400 | 0.018 | -0.117 | -0.011 |
|  | Knee position | 0.004 | 0.0127 | 0.284 | 0.777 | -0.022 | 0.029 |
|  | Ankle position | -0.069 | 0.0777 | -0.894 | 0.373 | -0.223 | 0.084 |

1. Random-effects meta-regression
2. Regression Based Test cannot be computed for subgroup(s) Population = Achilles tendinopathy, Stroke.
3. Standard error of effect size

**Supplementary file L. GRADE assessment per sub-group**

| **Subgroup** | **Risk of bias** | **Small sample bias** | **Inconsistency** | **Imprecision** | **Indirectness** | **Publication bias** | **Certainty** |
| --- | --- | --- | --- | --- | --- | --- | --- |
| **Healthy populations** | Downgrade | Do not downgrade (n=1,629) | Downgrade  (I^2^ = 100%). | Do not downgrade  (95% CI=89.8 to 92.6) | N/A | Downgrade  (Egger’s regression = -3.87, p<0.001) | Very low |
| **Achilles tendinopathy** | Downgrade | Downgrade  (n=35) | Downgrade  (I^2^ = 89%) | Downgrade  (95% CI=74.1 to 106.7) | N/A | N/A | Very low |
| **Stroke** | Downgrade | Downgrade  (n=36) | Do not downgrade  (I^2^ = 32%) | Downgrade  (95% CI=17.4 to 53.3) | N/A | N/A | Very low |
| *The certainty of the evidence was downgraded for risk of bias if >50% of studies were deemed as high risk of bias by the assessment of methodological quality criteria. The certainty of the evidence was downgraded for small sample bias when studies had <200 participants. The certainty of the evidence was downgraded for inconsistency if statistical heterogeneity (P<0.10 or I² ≥ 40%). The certainty of the evidence was downgraded for imprecision if wide confidence intervals or confidence intervals which crossed zero were present. The certainty of the evidence was downgraded for publication bias if the standard error coefficient in Egger’s test was significantly different from zero. Indirectness was not judged in this review as it was not relevant.* | | | | | | | |
